# Metal-like behavior of a 2D molecular catalyst enables redox-decoupled electrocatalysis

**DOI:** 10.1093/nsr/nwaf198

**Published:** 2025-05-20

**Authors:** Yang Wang, Dongyu Zhang, Ting Chen, Caijie Su, Yi Xie, Changzheng Wu, Nikolay Kornienko

**Affiliations:** State Key Laboratory of Precision and Intelligent Chemistry, University of Science and Technology of China, Hefei 230026, China; Institute of Inorganic Chemistry, Department of Chemistry, University of Bonn, Bonn 53121, Germany; Department of Chemical Engineering and Chemistry, Eindhoven University of Technology, Eindhoven 5612 AE, The Netherlands; State Key Laboratory of Precision and Intelligent Chemistry, University of Science and Technology of China, Hefei 230026, China; State Key Laboratory of Precision and Intelligent Chemistry, University of Science and Technology of China, Hefei 230026, China; State Key Laboratory of Precision and Intelligent Chemistry, University of Science and Technology of China, Hefei 230026, China; State Key Laboratory of Precision and Intelligent Chemistry, University of Science and Technology of China, Hefei 230026, China; Institute of Inorganic Chemistry, Department of Chemistry, University of Bonn, Bonn 53121, Germany

**Keywords:** molecular catalyst, two-dimensional confinement, metal-like electron transfer behavior, redox-decoupled mechanism, oxygen reduction reaction, fuel cells

## Abstract

Molecular catalysts facilitate electrochemical conversion by changing their oxidation states to transfer electrons. However, this redox-mediated mechanism features stepwise electron transfer and substrate activation in separate elementary steps, thereby resulting in an inherent loss in efficiency. Here, we synthesize a two-dimensional (2D) iron phthalocyanine (FePc) material and uncover its non-mediated electron transfer behavior in electrocatalysis, which overcomes the conventional redox-mediated limitation in the oxygen reduction reaction (ORR) pathway that molecular catalysts face. The 2D geometry enables the FePc molecules to be positioned within the electrochemical double layer, enabling electrons to directly transfer to oxygen reactants, prior to the Fe(II/III) redox. This functions in a manner akin to a metal catalyst thereby opening a redox-decoupled ORR mechanism. As a result, the reported 2D FePc molecular catalyst exhibits unprecedented ORR half-wave potential at 0.945 V vs. the reversible hydrogen electrode, achieving efficient application in zinc-air batteries and H_2_/O_2_ fuel cells. These findings open new possibilities in voltage efficient, redox-decoupled molecular catalysis that integrates strengths of molecules and materials in one synergistic system.

## Teaser

This study discovers that a two-dimensional molecular catalyst confined in the electrochemical double layer can behave like a metal, enabling efficient redox-decoupled electrocatalysis.

## INTRODUCTION

**Scheme 1.**
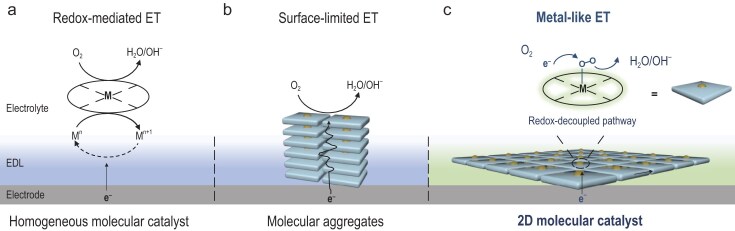
Electron transfer (ET) mechanism in molecular electrocatalysis. (a) Redox-mediated ET in homogeneous molecule systems. (b) Surface-limited ET in molecular aggregates systems. (c) Non-mediated ET in two-dimensional (2D) molecule systems, provided 2D molecules are positioned within the electrochemical double layer (EDL).

Electron transfer between electrodes and reactants is fundamental to electrochemical reaction, critically influencing the conversion efficiency of chemical to electrical energy in devices such as H_2_/O_2_ fuel cells [1] and metal-air batteries [2]. These electrocatalytic reactions typically occur at the metallic surface sites of heterogeneous electrocatalysts [3] or via redox mediation in homogeneous molecular electrocatalysts [4]. While conductive metal catalysts can continuously supply electrons without redox limitations, they usually lack defined active sites, making it difficult to attribute catalytic activity or selectivity to specific sites [5]. This ambiguity complicates the extrapolation of fundamental insights of catalytic mechanisms at the molecular level. Molecular systems are particularly attractive due to their ease of atomically precise structural tailoring, which allows for systematic tuning of selectivity and activity while providing unparalleled insights into reaction mechanisms [6,7]. In homogeneous molecular electrocatalysis [8], dissolved metal complexes shuttle between the electrode and electrolyte, transferring the electrons necessary for substrate activation (Scheme 1a). This process involves cycling between metal oxidation and reduction states, making electron transfer stepwise, non-infinite, non-direct, and non-continuous. Such a redox-mediated process often involves multiple complex reaction steps, thereby limiting the efficiency of electrocatalytic reactions. Additionally, the redox-coupled mechanism is limited by the voltage required to drive the redox transformation, while the redox-decoupled mechanism allows the reaction to proceed at less negative voltages. Recent studies have explored conjugated molecules such as metal phthalocyanines (MPcs) and metal porphyrins (MPors) to create heterogeneous molecular catalyst systems [9–11]. However, these systems typically exhibit ‘surface-limited electron transfer’ due to intrinsic intermolecular aggregation (Scheme 1b). In this context, ‘surface-limited electron transfer’ refers to the restriction of electron transfer to the outermost surface of the molecular aggregates, where the reactants can access. This limitation arises from the less conductive π-π stacked interlayers, which impede the efficient transfer of electrons through the bulk of the molecular aggregates [12–14].

Despite these challenges, molecular catalysts have long been hypothesized to enable more efficient electron transfer and higher reactivity, provided there is a strong enough electronic interaction between the molecule and the electrode [15]. Recently, Surendranath and colleagues established a series of graphite-conjugated molecular catalysts with strong electronic coupling to the electrode [16]. This resulted in concerted electron transfer behavior, where the oxidation state of the metal site remains unchanged. In a model system involving aliphatic linkages-assisted cobalt porphyrins adsorbed onto glassy carbon electrodes [17], they demonstrated that positioning the molecule within the electrochemical double layer (EDL) could eliminate the stepwise outer-sphere Co^II/I^ reduction-mediated hydrogen evolution reaction. Instead, it favored inner-sphere concerted proton-electron transfer pathways akin to those on metal surfaces [18]. These native surface interactions are likely generalizable to a wide range of metallo-macrocycle/carbon composite electrodes and represent a straightforward method to construct electronically coupled molecular electrodes [19,20]. However, confining molecules within the EDL at the electrode surface to achieve an electronically non-mediated, continuous behavior like a metal catalyst (‘metal-like’ behaviors) remains a significant challenge.

Two-dimensional (2D) materials, with their unique geometric features and electronic properties, present an ideal platform for surface catalysis [21]. Inspired by these materials, we hypothesized that confining active molecules within a 2D space could enhance their contact with the electrode, thereby strengthening electronic interactions. Additionally, this approach could facilitate the formation of extensive 2D networks of metallo-macrocycles linked by covalent bonds, preventing them from dissolving in aqueous solutions and ensuring their confinement within the EDL (Scheme 1c).

In this work, we developed a proof-of-concept study based on iron phthalocyanine (FePc) molecular model catalysts. We synthesized a 2D FePc molecular catalyst with each FePc molecule linked by covalent bonds into long-range ordered 2D networks. Due to its nanoscale thickness and microscale size, this 2D molecular catalyst is expected to reside in the EDL and create a strong electronic interaction with the conductive electrode (Scheme 1c). Interestingly, we observed a non-mediated electron transfer behavior on this 2D FePc molecular catalyst, opening an oxygen reduction reaction pathway preceding the redox reaction. Consequently, the ORR activity of the 2D FePc increased sharply, achieving a turnover frequency (TOF) that was several times higher than that of its redox-mediated 3D FePc counterparts, thus demonstrating exceptional potential for applications in both H_2_/O_2_ fuel cells and zinc-air batteries. Our results provide valuable insights for designing electron transfer in molecular electrocatalysis, especially for reactions involving multiple electron transfer steps.

## RESULTS AND DISCUSSION

### Synthesis and characterizations of 2D FePc molecular catalyst

**Figure 1.**
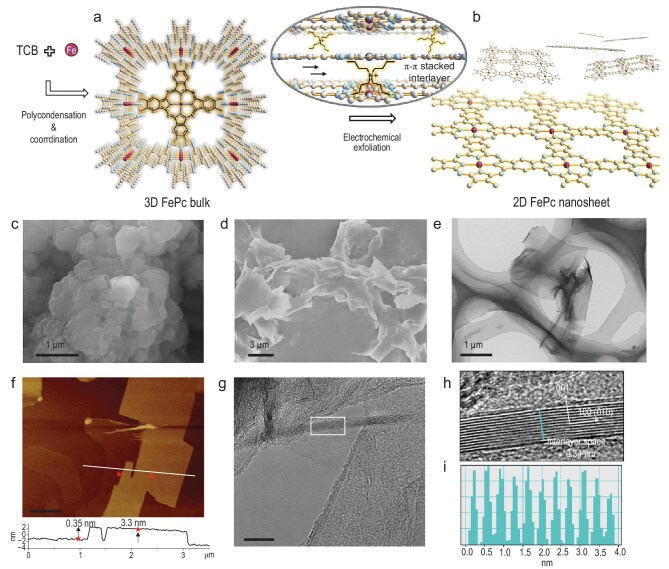
Synthesis and characterization of 2D FePc nanosheets. The 2D FePc nanosheets are synthesized with two steps. The first step was to synthesize 3D FePc bulk material via a polycondensation and coordination reaction (a), and then electrochemical exfoliation technology was employed to exfoliate π-π stacked layers into 2D FePc nanosheets (b). The 3D bulk materials display a layer-by-layer stacking morphology in the SEM image (c), while the 2D nanosheets exhibit thin, flaky structures in the SEM (d) and TEM (e) images typical of nanosheets. The resulting 2D nanosheets are ∼3.4 nm thick as shown in the AFM image (f) and feature an interlayer spacing of 0.34 nm in the HRTEM image and analysis (g–i). (h) is the magnification of the area marked by the rectangle in (g); (i) is analysed along the cyan line in (h). The scale bar is 500 nm for (f) and 10 nm for (g).

In this work, iron phthalocyanine (FePc) and its derivatives were prepared as model catalysts, due to their well-known reactivity in the oxygen reduction reaction (ORR). Moreover, their molecular-level structural precision and tuneability make them an ideal model system for gaining insight into electrocatalytic mechanisms [22]. As shown in Fig. 1a and b (see Methods and Synthesis scheme for details), this study first introduced a molecular construction strategy to synthesize a three-dimensional (3D) FePc bulk material. In this structure, individual FePc molecular units are covalently linked to form two-dimensional (2D) planar networks, which then self-assemble into a 3D bulk material (termed 3D FePc) through interlayer π-π stacking interactions [23]. To produce 2D nanosheets (termed 2D FePc), an electrochemical exfoliation strategy, developed in our previous work [24], was employed to break the layer-by-layer stacking of the 3D FePc aggregates. Specifically, the 3D bulk material was used as the cathode, while tetrabutylammonium bromide (TBA^+^) served as the intercalant. The intercalation process was performed at a working voltage of −5 V, where positively charged TBA^+^ ions, driven by the electric field, overcame the π-π interactions and intercalated between the 3D FePc layers, forming an intercalation compound. This compound was then transferred into a pure solvent, and 2D FePc nanosheets were obtained through simple manual shaking. The SEM image of the exfoliated 2D FePc material displayed a significant morphological change from the bulk material, showing thin, flaky structures typical of nanosheets (Fig. 1c, d). The TEM image in Fig. 1e further confirmed the sheet-like morphology, displaying micrometer-sized transparent regions indicative of a thin nanosheet structure with high surface area. The thicknesses of these nanosheets were further characterized by atomic force microscopy (AFM), as shown in Fig. 1f and Fig. S1. The nanosheets displayed a uniform size and thickness of 3–4 nm. The average thickness of the nanosheets was determined to be 3.44 nm with a standard deviation of 0.081 nm, indicating a structure consisting of only a few layers. A microscopic view of the layers can be seen in the HRTEM image, representing an interlayer space of 0.34 nm (Fig. 1g, h, i). Together, these distinct structural features of 2D FePc hold potential as electrocatalysts, providing greater exposure of active sites and facilitating more efficient contact with reagents compared to their 3D counterpart. The ultrathin thickness of nanosheets allows them to be confined within the EDL, which typically ranges from a few angstroms to tens of nanometers [25,26]. This was investigated by examining the electron transfer characteristics during the electrocatalysis reaction as discussed below.

Aside from exfoliation, the molecular structures of 2D FePc and 3D FePc remain almost unchanged, as characterized by FTIR and Raman spectroscopy (Figs S2 and S3), which showed identical peak positions. Similar to classical FePc molecules, the vibrational signal of 2D FePc at ∼745 cm^−1^ in the FTIR spectrum is attributed to coordinated Fe–N bonds that couple with the peripheral structures in the π-conjugated macrocycle, while a typical Fe–N–C local structure is also confirmed by Raman spectra appearing at 690 and 750 cm^−1^. This indicates successful coordination between Fe and N atoms, with the Fe-pyrrole N coordination environment further confirmed by XPS results (Fig. S4), which typically serves as ORR active sites consistent with iron coordinated tetrapyrrole in FePc. The weakened FTIR signals of 2D FePc compared to 3D FePc are mainly due to the thinning of stacked molecular layers after exfoliation. The FePc-based macrocycle structure was confirmed with the detection of a typical A_1__g_ signal at 1535 cm^−1^ and B_1__g_ signal at 1335 cm^−1^ (Fig. S3) [27]. Together, these data support the similarity in terms of molecular structures of the 2D FePc and its counterparts. Further, the diffraction for the (001) facet of eclipsed AA stacking of 3D FePc bulk material suggests structural ordering with an interlayer distance of ∼0.34 nm along the c axis [23]. A significant decrease in this intensity reflected that layer-by-layer π-π stacking was decreased through exfoliation (Fig. S5). The above consequences, i.e. the consistency of molecular structure and the change in crystallinity after exfoliation, support the fact that electrochemical exfoliation technology is effective and nondestructive, capable of generating a FePc material with 2D features without destroying its structure at the molecular scale. As a result, a well-defined 2D FePc molecule with precise iron coordinated tetrapyrrole active sites and 2D characteristics was obtained, providing an ideal model for further studies of ORR behavior.

**Figure 2.**
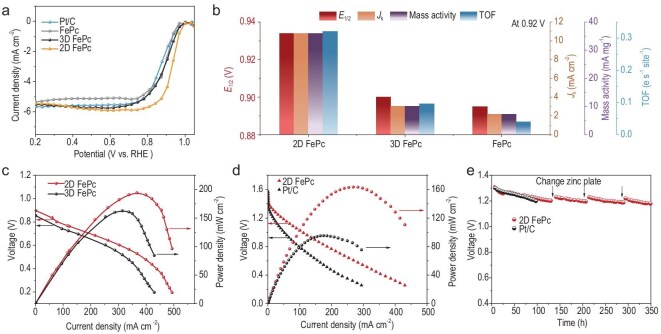
The comparison of ORR performance among 2D FePc, 3D FePc and classical FePc molecular catalysts (the loading of these three samples is 0.3 mg cm^−2^). (a) LSV curves, (b) a series of ORR performance parameters including half-wave potential (*E*_1/2_), kinetic current density (*J*_k_), mass activity and turnover frequency (TOF_Fe_) at the potential of 0.92 V point to the enhanced performance of the 2D FePc. The incorporation into a hydroxide exchange membrane fuel cell (c) and a zinc-air battery (d) showed the feasibility of the 2D FePc molecular catalyst in practical device applications. (e) Long-term discharge durability of 2D FePc in the zinc-air battery is far higher than that of a commercial Pt/C reference catalyst.

**Figure 3.**
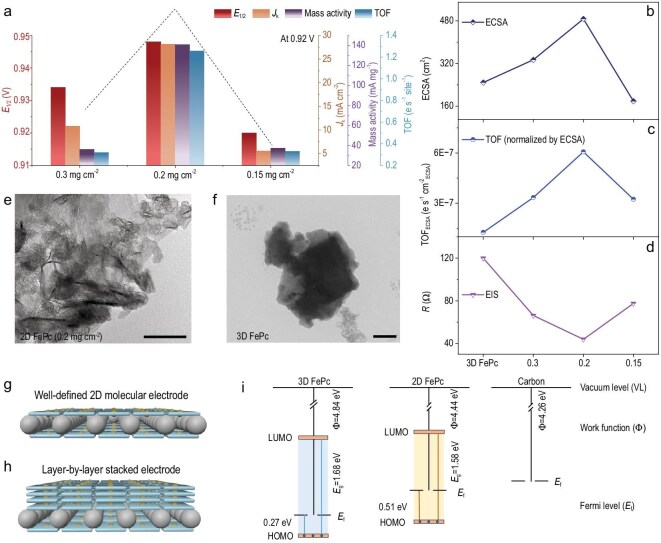
(a) The comparison of ORR performance among 2D FePc molecular catalysts with different loading of nanosheets, showed a nonlinear catalyst loading-activity relationship. The results of ECSA (b), TOF normalized by ECSA (c) and EIS (d) are displayed. The TEM images for (e) 2D FePc electrode with 0.2 mg cm^−2^ nanosheet loading (scale bar 100 nm) and (f) 3D FePc electrode (scale bar 100 nm) are also shown. Together with the above phenomenon, the scheme of well-defined 2D molecular electrode and layer-by-layer stacked electrode can be drawn in (g) and (h), respectively. The energy level structure (i) is shifted after exfoliation, enabling 2D FePc molecules to be more matched with the carbon electrode.

**Figure 4.**
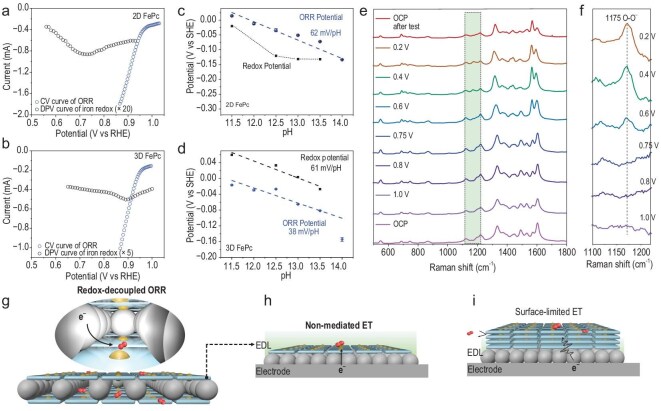
A redox-decoupled ORR mechanism via metal-like ET behavior. DPV curve (black) of iron redox and CV curve (blue) of ORR for 2D FePc molecular catalyst (a) and 3D FePc catalyst (b). Pourbaix diagram for 2D FePc molecular catalyst (c) and 3D FePc catalyst (d). (e) *In-situ* Raman spectra for 2D FePc molecular catalyst and (f) the zoomed-in view, using signal of different potentials minus OCP signal. The scheme of redox-decoupled ORR mechanism on the 2D FePc molecular catalyst (g), where the FePc molecule was positioned inside the EDL (h), while there was a surface-limited ET for the 3D FePc catalyst (i).

### ORR performance and applications in fuel cells and zinc-air batteries

The 2D FePc molecular catalyst holds particular advantages over both the normal FePc catalyst and the 3D FePc bulk material. Theoretically, the 2D structure has densely and regularly dispersed FePc molecule units, improving the utilization of active sites as compared to normal FePc aggregates. In addition, the long-range ordered planar structure enables active sites to be fully exposed, while the open 2D space and nanoscale thickness enhance mass transport, compared to the 3D bulk. In Fig. 2a, the linear sweep voltammetry (LSV) curves of ORR for these samples with the same catalyst loading (0.3 mg cm^−2^) exhibited significant differences, with the 2D FePc catalyst displaying a positive shift compared to FePc or 3D FePc control samples. The half-wave potential increased to 0.934 V, suggesting that the 2D FePc is superior to its aggregated counterparts. The similar limiting current of 2D and 3D FePc under RDE arises from identical oxygen diffusion rates caused by forced convection, reflecting diffusion-limited behavior rather than intrinsic activity. To reflect its intrinsic activity, the kinetic current density (*J*_k_), mass activity (MA) and turnover frequency of iron sites (TOF_Fe_) at 0.92 V potential are calculated and summarized in Fig. 2b and Fig. S6. The 2D FePc presented overall superiority in these electrochemical parameters. For example, the TOF displayed 10-fold and 5-fold enhancement compared to FePc and 3D FePc catalysts, respectively. In addition, electrochemical impedance spectroscopy (EIS) results indicated lower electron transfer resistance for the 2D molecular catalyst, with a 1.8-fold and 2.4-fold lower resistance than the 3D FePc and FePc, respectively (Fig. S14). These results together demonstrated more rapid reaction kinetics in the 2D molecular system. This is likely because electrons and reactants can easily be transported to the surface of the active site in the open 2D system, while in the 3D and normal FePc systems, they are hindered by poorly conductive π-π stacked interlayers, limiting their transport to the outermost surface for the reaction [10]. This surface-limited mass and electron transport hinders their performance for ORR. This speculation will be further validated in the following section by examining the relationship between loading and activity, as the 2D FePc loading decreases from 0.3 to 0.2 and 0.15 mg cm^−2^.

The stability of the 2D FePc catalyst was demonstrated through cyclic voltammogram (CV) cycling tests, and its key stability factors were confirmed by morphology retention (TEM), structural integrity (XRD), and chemical bond robustness (operando Raman), as shown in Fig. S15. The 2D FePc catalyst (0.2 mg cm^−2^) exhibited exceptional stability, enduring 10 000 and 20 000 CV cycles with minimal half-wave potential decays of only 8 mV and 12 mV, respectively. TEM images reveal that the morphology of the 2D FePc catalyst remains nearly unchanged, maintaining a homogeneous mixture of carbon black and nanosheets without observable aggregation. This suggests that the 2D FePc/carbon electrode configuration remains intact, ensuring the active sites stay immobilized within the EDL. The XRD pattern of 2D FePc after 20 000 cycles exhibits no significant changes or any signals of metal particles, further confirming the structural integrity and stability of its Fe-N_4_ active sites. In addition, the operando Raman spectra display peaks at ∼690 and ∼750 cm^−1^, associated with the localized Fe–N coordination structure, which persist after extended cycling. This indicates that the 2D confinement of FePc molecular catalyst, stabilized by fully conjugated covalent bonds, effectively enhances the robustness of the core Fe-N_4_ structure.

Benefiting from its outstanding ORR performance, the 2D FePc molecular catalyst was assembled into a membrane electrode and a gas diffusion electrode for practical application in hydroxide exchange membrane fuel cells (HEMFCs) and zinc-air batteries, respectively. In HEMFC single cell tests, the 2D FePc outperformed its 3D FePc counterpart, achieving a peak power density approaching 200 mW cm^−2^. Moreover, the 2D FePc displayed a higher starting voltage in the polarization curve, reflecting its superior intrinsic activity corresponding to its ORR activity in RDE tests (Fig. 2c). Meanwhile, the 2D FePc molecular catalyst in the zinc-air battery delivered a peak power density of 165 mW cm^−2^ at ∼0.6 V with a current density of 270 mA cm^−2^, nearly twice that of the benchmarking commercial Pt/C catalyst used in an equivalent setup (Fig. 2d). The polarization behavior of the HEMFC and zinc-air battery devices can be divided into activation, ohmic, and concentration polarization regions. In the high current density region (>300 mA cm^−2^), a noticeable voltage drop is observed, which can be primarily attributed to oxygen mass transport limitations. Importantly, for the 2D FePc electrode, this voltage drop occurs at a higher current density (>400 mA cm^−2^), suggesting improved mass transport due to the well-dispersed nanosheet/carbon composite structure. In the zinc-air battery configuration, the 2D FePc electrode maintains a stable slope even at >350 mA cm^−2^, further confirming its efficient mass transport under high-load conditions. In addition, during long-term discharge tests, the 2D FePc molecular catalyst demonstrated high durability, lasting up to 350 hours discharge with <10% decay, while the Pt/C could only undergo 100 hours of continual testing (Fig. 2e). To further substantiate the structural stability of the 2D FePc electrode under practical operating conditions, we conducted additional TEM characterizations after both the HEMFC and zinc-air battery tests (Fig. S16). The post-test TEM images reveal that the overall morphology of the 2D FePc structure is largely preserved, indicating that the material maintains good dispersion and structural integrity during practical operation.

Together, these results support the notion that the design of the 2D structure enabled the FePc molecule to overcome limitations in active site exposure and mass transport, leading to significant enhancements in ORR electrocatalysis. Compared to most Fe-N-C catalysts summarized in Table S1 and start-of-the-art ORR catalyst in Table S2, the 2D FePc molecular catalyst demonstrates superior performance.

### Electron transfer behavior

To further investigate how the 2D characteristics of the molecular catalyst influence ORR behavior, we conducted activity studies related to 2D nanosheet loading. During the electrode preparation, nanosheets and conductive carbon black were mixed in different ratios to achieve 2D FePc molecular loadings of 0.3, 0.2, and 0.15 mg cm^−2^. Their ORR activity was tested (Fig. S7) and summarized in Fig. 3a. Typically, there exists a linear relationship between loading and activity, where an increase in loading usually translates to more active sites available for catalyzing reactions, thereby enhancing the ORR activity. However, Fig. 3a reveals a nonlinear loading-activity relationship for the 2D molecular catalyst system. When the loading increased from 0.15 to 0.2 mg cm^−2^, there was a typical rise in activity. However, further increasing the loading to 0.3 mg cm^−2^ reversed this trend. The highest ORR activity was observed at a 0.2 mg cm^−2^ loading, with a half-wave potential of 0.945 V, which is 25 mV higher than the 0.15 mg cm^−2^ loading and 11 mV higher than the 0.3 mg cm^−2^ loading.

This unusual phenomenon suggests that the number of exposed active sites actually involved in the ORR reaction did not increase with the loading from 0.2 to 0.3 mg cm^−2^. Surprisingly, the electrochemcial active surface area (ECSA) results (Fig. 3b and Fig. S8) similarly showed a decreasing trend from 0.2 to 0.3 mg cm^−2^ loading. The ECSA value for the 2D FePc catalyst with a 0.2 mg cm^−2^ loading was calculated from double layer capacitance (3.82 mF), nearly 1.5 times and 2.0 times that of the 0.3 mg cm^−2^ loading and 3D FePc, respectively. Moreover, TOF normalized by ECSA (TOF_ECSA_) was calculated to provide a more precise comparison of intrinsic activity per effective active site (Fig. 3c). Despite the increased ECSA, the TOF_ECSA_ for the optimal 2D FePc (0.2 mg cm^−2^) catalyst remains 1.8 and 4.7 times higher than that of another 2D FePc (0.3 mg cm^−2^) and 3D FePc catalyst, respectively, demonstrating its superior intrinsic activity. Electrochemical impedance spectroscopy (EIS) data (Fig. 3d and Fig. S14) and Tafel slope results (Fig. S9a) also supported this phenomenon, reflecting superior charge transfer and faster ORR kinetics. In addition to its high activity, 2D FePc (0.2 mg cm^−2^) demonstrates exceptional 4e^−^ selectivity and an ultralow H_2_O_2_ yield, indicating highly efficient electron utilization (Fig. S9b).

These results indicate that the performance of a 2D molecular catalyst electrode is maximized by improving active site exposure and accelerating electron transfer. This optimization is achieved when the thin 2D FePc nanosheets are well-dispersed on the electrode surface. In the 0.2 mg cm^−2^ loading system (Fig. 3e), the catalyst forms a homogeneous hybrid with the carbon substrate, preventing aggregation into thicker layers, as schematically illustrated in Fig. 3g. Conversely, increasing the nanosheet loading to 0.3 mg cm^−2^ probably triggers self-aggregation due to intrinsic π-π interactions. Similar to 3D FePc bulk (Fig. 3f), these interactions would result in poor dispersion (Fig. S10), which thereby manifests itself as a reduced ECSA. This is depicted in Fig. 3h. On the other hand, a catalyst loading that has been slightly decreased to 0.15 mg cm^−2^ possibly leads to an aggregation of the carbon black and consequently leads to FePc layer aggregation as well (Fig. S10). This also results in lower metrics like ECSA, MA and TOF. These findings highlight that only an optimized catalyst-to-carbon ratio allows for an effective integration and dispersion of both components, yielding a well-defined electrode architecture. This precisely engineered 2D molecular electrode allows ultrathin molecular layers to intimately adhere to the uniformly distributed carbon black surface, forming a conductive and well-integrated electrode (Fig. 3g). In this configuration, the electron transfer from carbon substrates to active sites is likely to be both direct and efficient.

To demonstrate the potential mechanism of electron transfer behavior, we further studied the interfacial energy level structure. Figure 3i shows the energy level diagram of 3D FePc bulk material and 2D FePc nanosheet, in which the data are extracted from synchrotron radiation photoelectron spectra (SRPES) in Fig. S11 and ultraviolet visible electronic absorption spectroscopy (UV-VIS) in Fig. S12. Reducing the thickness of the stacked molecular layers shifts the overall energy level upward and narrows the gap between the highest occupied molecular orbital (HOMO) and the lowest unoccupied molecular orbital (LUMO). The energy shift did not cause the obvious change of Fe valence and XPS data showed the mixed valence character of Fe with the ratio of Fe^3+^ and Fe^2+^ being almost the same (Fig. S13). Additionally, the Fermi level of 2D FePc is closer to that of the carbon substrate, indicating an energetically better matched interface. As a result, the interfacial electron transfer dynamics would theoretically be fast and the electrons will transfer from higher Fermi level (carbon) to lower Fermi level (2D FePc) [28,29]. Moreover, this reduction in the HOMO-LUMO energy gap implies that electrons require less energy to transition, facilitating faster electron transfer kinetics. EIS fitting results (Fig. 3d and Fig. S14) confirmed a nearly 3-fold lower charge transfer resistance in the 2D FePc with 0.2 mg cm^−2^ loading as compared to its 3D counterpart. This direct and rapid electron transfer significantly accelerated ORR kinetics on the iron site in 2D FePc molecular catalyst as compared to the 3D FePc, which is reflected in the TOF data.

As a result, these data support that a well-defined 2D molecular catalyst electrode can be obtained through a simple method of adjusting the loading of nanosheets, demonstrating the superiority of the 2D material system. It is reasonable to believe that this enhanced electron transfer behavior is an intrinsic characteristic of the 2D molecular catalyst system due to more accessible contact and stronger electronic interaction between the 2D layers and conductive carbon support.

### Redox-decoupled mechanism

The well-defined nature of the 2D molecular catalyst in our work facilitates the elucidation of key mechanistic aspects. Conventionally, molecular catalysts follow a redox-mediated ORR mechanism, where dissolved molecules shuttle between the electrolyte and electrode to transfer electrons, accompanied by a change in the metal oxidation state (e.g. Fe^3+^/Fe^2+^) that then enables the catalytic reaction to be carried out [4]. In this 2D molecular catalyst system, however, the FePc molecules are well integrated into conductive electrodes. During electrocatalysis, the 2D sheets are immobilized within the EDL and intimately adhere to the carbon support, which may enable them to behave partly like a conductive metal catalyst and carry out the ORR reaction without first needing to be reduced to Fe^2+^ (e.g. redox-decoupled). For ease of understanding, this section will focus on two representative molecular catalyst systems, 2D FePc (0.2 mg cm^−2^) and 3D FePc catalyst.

First, as indicated by the CV test, the redox characteristics of the catalytic system change with the dimensionality of the catalyst. For the 2D molecular catalyst with a 0.2 mg cm^−2^ loading, there is a pair of redox peaks in 0.1 M KOH under N_2_ atmosphere (Fig. S17). Through the comparison of CV curves scanning at different potential ranges, we found that the reduction peak at ∼0.75 V and oxidation peak at ∼1.0 V are a pair of coupled peaks, appearing and disappearing synchronously. Moreover, this redox peak showed good reversibility when tested at different scan rates (Fig. S18). These results confirmed that the redox peaks belong to Fe^3+^/Fe^2+^ reduction and Fe^2+^/Fe^3+^ oxidation [30]. Furthermore, differential pulse voltammogram (DPV) technology was employed to provide more accurate information on the Fe^3+^/Fe^2+^ reduction potential. For 2D FePc, this peak emerged at 0.72 V, more negative than the experimentally measured ORR onset potential (Fig. 4a). In contrast, for the 3D FePc, the Fe^3+^/Fe^2+^ reduction peak at ∼0.90 V occurred together with the onset of ORR (Fig. 4b and Fig. S19). On the one hand, the difference in redox potential may be ascribed to the energy level upshift of molecules as confirmed in Fig. 3i. In the 2D conjugated networks, the d-orbital electrons of Fe can conjugate with delocalized π-electrons of the macrocyclic skeleton [31,32]. Compared to 3D FePc, this upshifted energy level suggests that electrons must overcome a higher energy barrier before being injected into the 2D FePc molecule. As a result, a more negative voltage is necessary to supply electrons for the reduction of Fe^3+^ to Fe^2+^. On the other hand, the energy level alignment indicates that 2D FePc has a better electronic compatibility with the carbon substrate, probably resulting in stronger electronic coupling between them. This interfacial interaction may enable the 2D FePc molecules to better integrate electronically with the carbon substrate, effectively functioning as a hybrid system where the molecular energy levels merge into the band structure of the conductive substrate [16]. Under these conditions, the molecular catalyst behaves as a hybrid electrocatalyst, with its potential partially modulated by the electrode [15]. This shift in redox potential would induce distinct ORR mechanisms between the two catalysts. During ORR, 3D FePc follows a redox-mediated behavior. Conversely, the well-defined 2D FePc molecular catalyst is able to bypass the redox-mediated pathway with its unchanged Fe valence, when working at high potential (>0.75 V). A direct and efficient electron transfer in this voltage range likely leads to a redox-decoupled ORR mechanism.

The redox and ORR behavior of both samples was tested at various pH values within the alkaline range. As shown in Fig. 4c and Fig. S20, the ORR potential versus SHE at the same certain current (0.1 mA) showed a linear relationship with pH, with a fitting slope of 62 mV/pH for the 2D FePc, which aligns with the theoretical Nernstian scaling of 59 mV/pH. In contrast, the Fe^3+^/Fe^2+^ redox midpoint potential, here quantified as the midpoint between the reductive and oxidative waves of the Fe^3+^/Fe^2+^ couple, is located negative to the ORR potential (Fig. 4c and Fig. S21). These results suggest that the ORR is not mediated by the Fe^3+^/Fe^2+^ couple, when the applied voltage is higher than the redox potential (>0.75 V). As a result, a redox-decoupled, proton-electron concerted reaction sequence is suggested to operate for the 2D FePc molecule, instead of the redox-mediated stepwise sequence typical for molecular catalysts [17]. This probably steps from the well-dispersed 2D molecular layers positioned inside the EDL on the electrode's surface (schemed in Fig. 4g and h). Here a ‘metal-like’ electron transfer behavior is triggered to enable direct O_2_ reduction at >0.75 V applied potential. However, for the 3D FePc catalyst (Fig. 4d and Fig. S22), the redox potential is positive to ORR potential, suggesting the redox of the Fe^3+^/Fe^2+^ couple proceeding in ORR. Linear fitting shows a slope of 38 mV/pH for ORR, which deviates significantly from the expected 59 mV/pH, indicating a non-standard proton-coupled electron transfer (PCET) process. The slope of 61 mV/pH for its redox potential suggests that the redox process of Fe^3+^/Fe^2+^ is coupled with the release of OH^−^ ions (Fig. 4d and Fig. S23) [30]. This observation is characteristic of conventional FePc catalysts, where the reduction of Fe^3+^ to Fe^2+^ is accompanied by the detachment of one OH⁻ ion [33,34]. This is because the stacked layers in a 3D FePc catalyst exceed the thickness of EDL, resulting in a low efficient surface-limited electron transfer. Electrons must traverse the poorly conductive interlayer spaces before being utilized for O_2_ reduction, alongside the synchronous reduction of Fe^3+^. Together considering the overlap of reduction potential of Fe^3+^ and O_2_, the 3D FePc catalyst exhibits a surface-limited electron transfer-induced redox-mediated ORR mechanism (as schemed in Fig. 4i). Consequently, significant differences in reaction kinetics are observed between the redox-decoupled mechanism of 2D FePc and the redox-mediated mechanism of 3D FePc, as evidenced by the Tafel slopes (Fig. S9).

To add visible evidence supporting the aforementioned mechanism, we have performed in-situ Raman spectroscopy to detect key intermediates that distinguish the redox-decoupled mechanism from the redox-mediated one. In the redox-mediated ORR mechanism, it has been reported that the Fe^3+^ is initially reduced to Fe^2+^. The Fe^2+^ site subsequently donates an electron to the adsorbed O_2_, forming Fe^3+^–OO^−^ intermediate in alkaline electrolytes [35,36]. In contrast, in the PCET mechanism, one proton and one electron are transferred simultaneously to the adsorbed O_2_, directly forming Fe^3+^–OOH intermediate, which rapidly transforms into subsequent *O and *OH species. It is noted that these intermediates are detectable via Raman spectroscopy only when their concentrations are sufficient. In the *in-situ* Raman spectra for the 2D FePc molecular catalyst (Fig. 4e and f), only the signal corresponding to superoxide species (O_2_^−^) is observed at ∼1175 cm^−1^, and this signal varies with applied potential. Importantly, this signal is observed only at potentials below 0.75 V, suggesting that a redox-mediated O_2_ reduction process takes place at these lower potentials, consistent with the Fe^3+^/Fe^2+^ redox potential. However, when the applied potential exceeds 0.75 V, the O_2_^−^ signal is not observed. Above this potential, the Fe redox couple remains unreduced (e.g. remains in the Fe^3+^ state). This observation suggests that the redox-decoupled PCET process is dominant at higher potentials. These electrochemical analysis and *in-situ* spectroscopy together support that downscaling to 2D dimensions enables a redox-decoupled ORR preceding redox potential on FePc molecular catalyst.

Moreover, some recent work [15] corroborate our findings in an indirect manner, highlighting the significance of electrochemical efficiency through immobilizing active molecules within the EDL, underscoring the generality and scalability of our approach.

## CONCLUSION

A 2D space confinement strategy was introduced to extend iron phthalocyanine (FePc) molecules into 2D molecular networks. These networks are fixed within the EDL, ensuring intimate contact and electronic interaction with the electrode's surface. Electrochemical characterization and *in-situ* Raman spectroscopy reveal a non-mediated electron transfer behavior in the 2D FePc layers at high potential. This enables a redox-decoupled ORR mechanism, unlike the redox-mediated mechanism observed in the 3D FePc material. Consequently, the 2D FePc catalyst delivers an unprecedented ORR activity, thus achieving a peak power density approaching 200 mW cm^−2^ in H_2_/O_2_ fuel cells and sustaining stable discharge for two weeks in zinc-air batteries. These findings underscore the potential of 2D molecular catalysts to combine molecular precision with efficient electron transfer and electronic integration with conductive electrodes, offering valuable insights towards the design of next generation electrocatalysts. This approach is particularly promising for reactions involving multiple electron transfer steps, such as the conversion of carbon dioxide to high-value chemicals.

## MATERIALS AND METHODS

See details in the Supplementary data.

## Supplementary Material

nwaf198_Supplemental_File
