# Supplementary material for: Metal-like behavior of a 2D molecular catalyst enables redox-decoupled electrocatalysis
Source: Natl Sci Rev. 2025 May 20;12(8):nwaf198. doi: 10.1093/nsr/nwaf198 (PMC12236314; doi:10.1093/nsr/nwaf198)
Supplement: nwaf198_Supplemental_File [file nwaf198_supplemental_file.pdf]

Supporting Information

Metal-like Behavior of a 2D Molecular Catalyst Enables Redox-Decoupled Electrocatalysis

Yang Wang[1a,b], Dongyu Zhang[1c], Ting Chen[1a], Caijie Su[a], Yi Xie[a], Changzheng Wu*[,a], Nikolay Kornienko*[,b]

[a] State Key Laboratory of Precision and Intelligent Chemistry, University of Science and Technology of China, Hefei, Anhui 230026, China.

[b] Institute of Inorganic Chemistry, University of Bonn, Gerhard-Domagk-Strasse 1, 53121 Bonn, Germany.

[c] Department of Chemical Engineering and Chemistry, Eindhoven University of Technology, Groene Loper 3, Eindhoven 5612 AE, The Netherlands.

[1]These authors contributed equally: Yang Wang, Dongyu Zhang and Ting Chen.

* Correspondence: czwu@ustc.edu.cn and nkornien@uni-bonn.de.

Methods

Synthesis of 3D FePc bulk material: The 3D FePc bulk material was synthesized using our previously reported method with minor modifications[1]. A 10 mL high-pressure Schlenk tube was charged with Benzene-1,2,4,5-tetracarbonitrile (1 mmol, 178 mg), anhydrous $FeCl_2$ (0.5mmol, 64mg), DBU (7.34 mg, 0.048 mmol) and 2 mL n-pentanol solvent in a glove box. The tube was sealed and sonicated at room temperature for 10 min, degassed by three freeze-pump-thaw cycles, sealed under vacuum and heated at 160 °C for three days. After cooling to room temperature, the mixture was filtrated, washed with methanol. After Soxhlet extraction with methanol (24 h) and acetone (24 h), 3D FePc bulk material was collected and dried under vacuum at 120 °C overnight to get dark green powders. The Fe content of the 3D FePc was determined to be 6.1 wt% using an inductively coupled plasma-atomic emission spectrometer.

Synthesis of 2D FePc nanosheets: The 2D FePc nanosheets were synthesized using our previously reported method with minor modifications[2]. The as-synthesized 3D FePc bulk material was used as precursors for the preparation of nanosheets via intercalation in a two-electrode electrochemical cell. The bulk material was anchored between two titanium as the cathode, with a platinum plate serving as the anode. The electrolyte contained 0.01 M tetrabutylammonium bromide (TBA^+) in acetonitrile. The intercalation process was carried out at a voltage of -5 V for a duration of 1 hour. During this process, positively charged TBA^+ ions driven by the electric field can intercalate between the layers of FePc bulk, forming an intercalation compound. Subsequently, the intercalation compound was transferred into methanol solvent, and 2D FePc nanosheets in solution were obtained by simply manual shaking. To remove large chunks and impurities, the product was purified by multiple cycles of washing and centrifugation. The Fe content of the 2D FePc was determined to be 6.49 wt% using an inductively coupled plasma-atomic emission spectrometer.

Synthesis of FePc molecule: The FePc molecule was synthesized using our previously reported method[3]. Phthalonitrile (10 mmol, 1.28 g) fragments were ground and evenly mixed with urea (80 mmol, 4.80 g), anhydrous $FeCl_2$ (2.5 mmol, 0.32 g) and trace amount of $(NH_4)_2MoO_4$, and then

reacted at 200 °C under argon atmosphere for 8 hours. After cooling down to room temperature, the crude product was stirred in 1 M HCl solution for 1 h. The solid was filtered and then stirred in 1 M NaOH solution for 1 h. The crude product was filtered, washed with deionized water, dried in vacuum. After Soxhlet extraction with methanol (24 h) and acetone (24 h), FePc molecule was collected and dried under vacuum at 120 °C overnight to get dark green powders. The Fe content of the FePc molecule was determined to be 10.6 wt% using an inductively coupled plasma-atomic emission spectrometer.

Electrochemical Measurements. The electrochemical tests were conducted with a three-electrode test mode on an electrochemical workstation. A glassy carbon (GC) electrode (diameter of 5 mm with surface area of 0.196 cm^2) was used as the working electrode, while a graphite rod was used as the counter electrode. The saturated Ag/AgCl electrode was used as reference, calculating RHE with E_{RHE} (V) = $E_{Ag/AgCl}$ + 0.0592 pH + 0.197. In regards of preparing working electrode, 4 mg FePc-based molecule/carbon black hybrids with a range of ratio and 40 μl Nafion solution (Sigma Aldrich, 5wt %) were dispersed in 1 ml of water-isopropanol solution with volume ratio of 3:1 by sonicating for 60 min to form a homogeneous ink. The ink was then drop-casted onto the glassy carbon electrode with a 0.6 mg cm^{-2} total loading for all samples. FePc and 3D FePc catalyst hybrids are prepared with 1:1 (molecule/carbon) ratio (0.3 mg cm^{-2} loading), while 2D FePc catalysts with 0.3, 0.2 and 0.15 mg cm^{-2} loading are prepared with 1:1, 1:2 and 1:3 mass ratio, respectively. Moreover, in this different ratio, the mass of carbon is the same. Oxygen was used to purge the 0.1 M KOH solution for 30 min to keep the solution oxygen saturation before ORR testing. Before cyclic voltammetry (CV), linear sweep voltammetry (LSV) and differential pulse voltammetry (DPV) tests, the working electrodes were fully activated using CV cycling for about 20 mins till the curves overlapped. The CV and LSV were recorded at a scan rate of 50 mV s^{-1} and 10 mV s^{-1}, respectively. The rotation rate of the RDE is set at 1600 rpm unless otherwise specified. The kinetic current densities (J_k) were calculated according to Koutecky-Levich equation, at different electrode potentials:

$$\frac{1}{J} = \frac{1}{J_k} + \frac{1}{J_L} = \frac{1}{J_k} + \frac{1}{Bw^{1/2}} \qquad \text{equation 1}$$

$$\mathrm{B} = 0.62\mathrm{nF}C_0 D_0^{2/3} v^{-1/6} \qquad \text{equation 2}$$

Where J is the measured current density, J_L is diffusion-limiting current density, w is the angular velocity, F is the Faraday constant (96485 C mol^{-1}), C_0 is the bulk concentration of O_2 (1.2 × 10^{-6} mol cm^{-3}), D_0 is the diffusion coefficient of O_2 in 0.1 M KOH (1.9 × 10^{-5} cm^2 s^{-1}) and v is the kinematic viscosity of the electrolyte (0.01 cm^2 s^{-1}).

The mass activity (MA) of catalytic molecule was calculated according to the equations:

$$MA = \frac{J_k}{m_{mole}} \qquad \text{equation 3}$$

The electrochemical surface area (ECSA) of catalyst was calculated according to the equation:

$$C_{dl} = \frac{Q}{U} = \frac{dQ/dt}{dU/dt} = \frac{J}{r} \qquad \text{equation 4}$$

$$ECSA = \frac{C_{dl}}{C_s} \qquad \text{equation 5}$$

where Q is the quantity of electric charge per unit area, U is the voltage, J is the current density and r is the scan rate, so the double-layer capacitance (C_{dl}) is the slope of J~r. C_s is the specific capacitance value for a flat standard with 1 cm^2 of real surface area. Here C_s is the average value (40 $\mu F \cdot cm^{-2}$).[4]

The turnover frequency (TOF) of catalytic Fe sites were calculated according to two methods:

In order to easily compare 2D FePc and 3D FePc catalyst, the first method regards all Fe atom as effective active sites. The equation is:

$$TOF_{Fe} = \frac{J_k N_e}{\omega m_{mole} N_A / M} \qquad \text{equation 6}$$

where J_k is the kinetic current density, N_e is the electron number per Coulomb (6.24×10^{18}), ω is the content of Fe sites in the catalyst, m_{mole} is the loading of FePc-based molecule, N_A is the Avogadro constant (6.022×10^{23}), and M is the mass per mole of Fe.[5]

The second formula is to precisely compare activity of per effective active site, using the electrochemical surface area (ECSA) for a practical reflection of the active sites. As a result, the TOF normalized by ECSA is simplified with the formula:

$$TOF_{ECSA} = \frac{J_k N_e}{ECSA N_A} \qquad \text{equation 7}$$

Hydroxide Exchange Membrane Fuel cell (HEMFC) tests. Hydroxide exchange membrane fuel cell (HEMFC) single cell was driven by 2D FePc catalyst and control samples (3D FePc) as cathode catalyst, PtRu/C as anode catalyst and commercial FAA-3-50 anion exchange membrane (FuMA-Tech) as hydroxide exchange membrane (HEM). The corresponding gas diffusion electrodes (GDEs) were prepared according to our previous literature[6]. Firstly, the obtained catalyst ink was sprayed on a carbon paper (Toray TGP-H-60) to fabricate gas diffusion electrode (GDE with an electrode area of 6.25 cm^2). The catalyst (2D FePc/3D FePc : Ketjen carbon black = 1:2/1:1) loadings in electrode was 1 mg cm^{-2}. The GDEs and HEM were then converted to OH^- form by immersing in aqueous NaOH (1 M) solution for 12 h followed by thoroughly washing with DI water. Finally, membrane electrode assembly (MEA) was fabricated by sandwiching the HEM between the cathode and anode GDEs with no hot-pressing used. An 850C fuel cell test station (Scribner Associates, USA) was used for testing the H_2/O_2 single cell performance of the as-prepared MEA using galvanostatic discharge steps. The H_2/O_2 gases were humidified (100 RH %) and supplied to the cell without backpressure at 60 °C temperature.

Zinc-air battery tests. The homemade Zn-air battery stack was assembled according to the following steps: 6 M KOH was used as electrolyte and a polished zinc foil was used as the anode. Typically, the air electrode was fabricated by spraying catalyst ink onto the gas diffusion layer (purchased from Youteke Changzhou). On average, 3 mg 2D molecular catalyst (1 mg 2D FePc molecule mixed with 2 mg carbon black) or 3 mg commercial Pt/C mixed with 40 μl Nafion solution (Sigma Aldrich, 5 wt%) were dispersed in 0.5 ml of water/isopropanol solution. The electrocatalyst loading of air electrode is 1 mg cm^{-2}. The test was conducted in air conditions without pumping oxygen.

Other Characterizations. Powder X-ray diffusion (PXRD) was performed on a Philips X'Pert Pro Super diffractometer with Cu Kα radiation (λ = 1.54178 Å). SEM images were acquired on a Carl Zeiss Supra 40. High resolution transmission electron microscope (HRTEM) images were tested on

a JEOL JEM-ARF200F transmission/scanning transmission electron microscope with a spherical aberration corrector. Fourier Transform Infrared spectroscopy (FTIR) was recorded on a Thermo Nicolet FTIR spectrometer. Raman spectra was recorded on a LabRAM-HR Confocal Laser Micro Raman Spectrometer with excitation wavelength of 532 nm. Ultraviolet visible light spectrum (UV-VIS) was measured using a Shimadzu SolidSpec-3700. X-ray photoelectron spectrometer (XPS) measurements were carried out on an ESCALAB MK II X-ray photoelectron spectrometer with Mg-Ka as the excitation source. The loading content of Fe was measured on an inductively coupled plasma-atomic emission spectrometer (ICP-AES) by employing an Optima 7300 DV (PerkinElmer). Synchrotron radiation photoelectron spectra (SRPES) experiments and soft X-ray absorption near-edge structure (XANES) were carried out at the Catalysis and Surface Science Endstation at the BL11U beamline in the National Synchrotron Radiation Laboratory (NSRL) in Hefei, China.

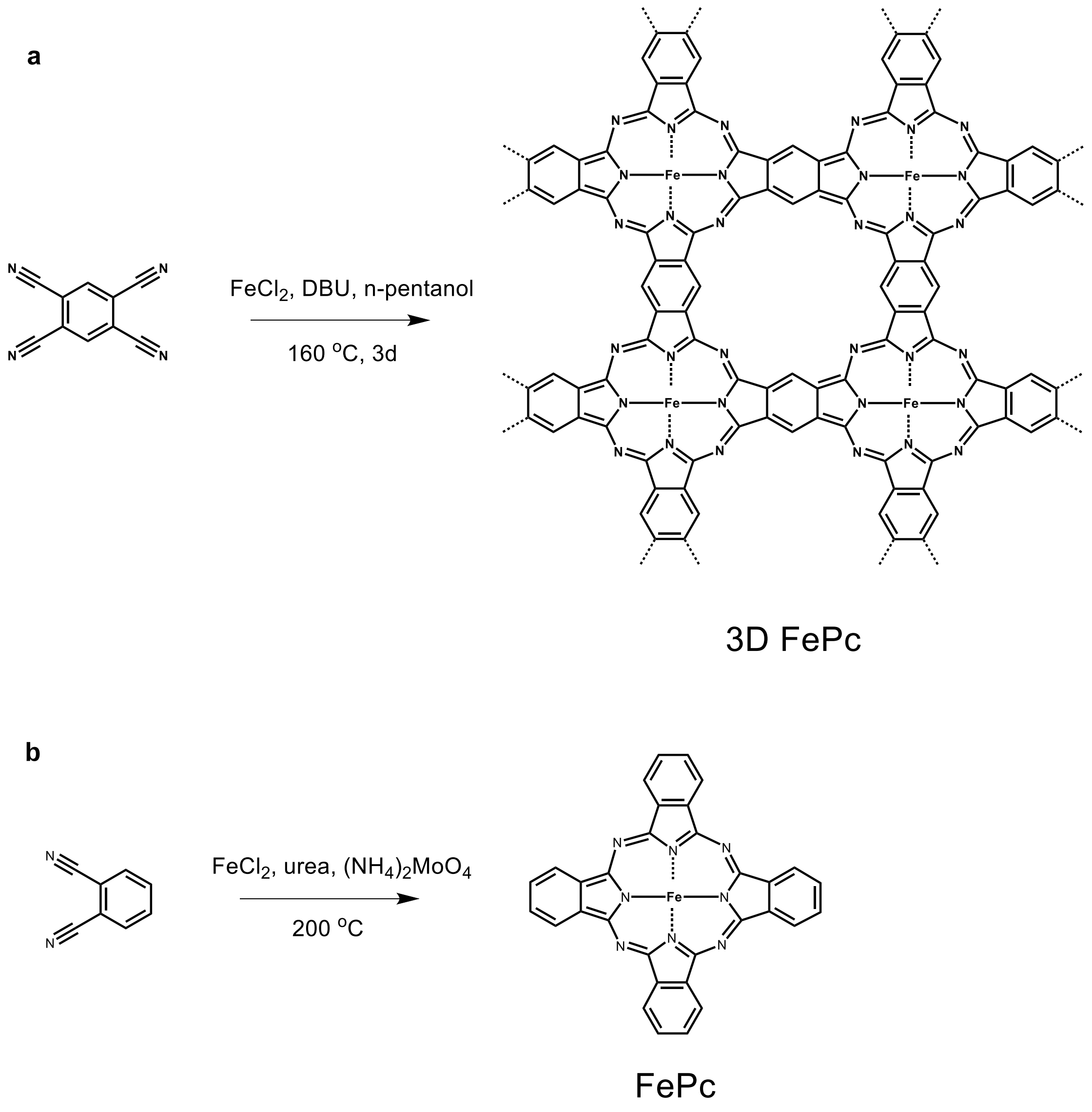

Synthesis scheme. The synthesis routes for (a) 3D FePc bulk material and (b) FePc molecule.

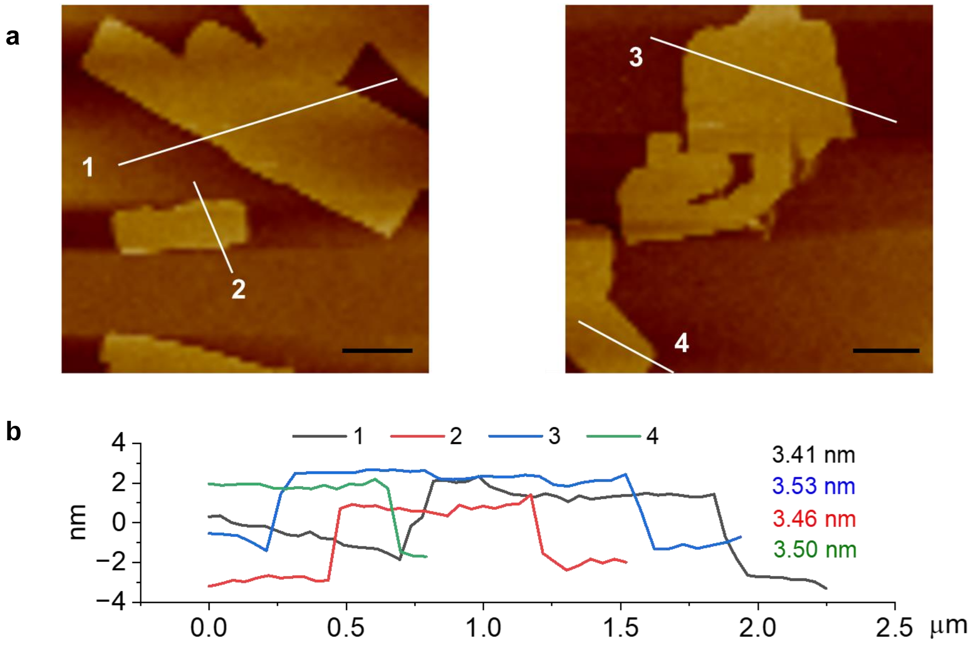

Figure S1. AFM images for 2D FePc nanosheets. The tested height of different nanosheets (a) are shown in (b). The scale bar is 500 nm.

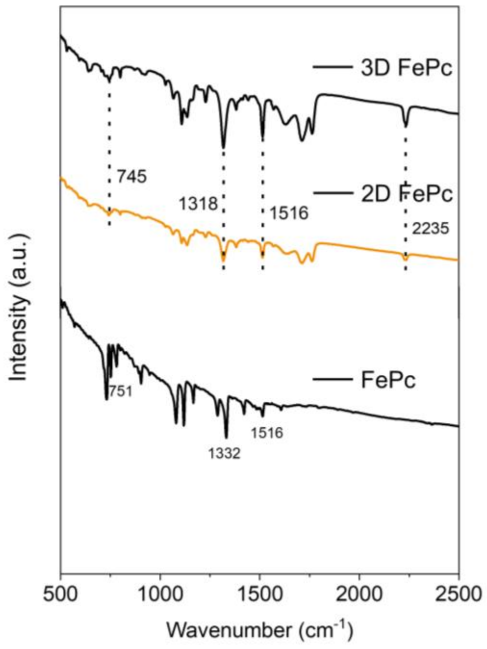

Figure S2. Fourier transform infrared spectra for 3D FePc, 2D FePc and FePc.

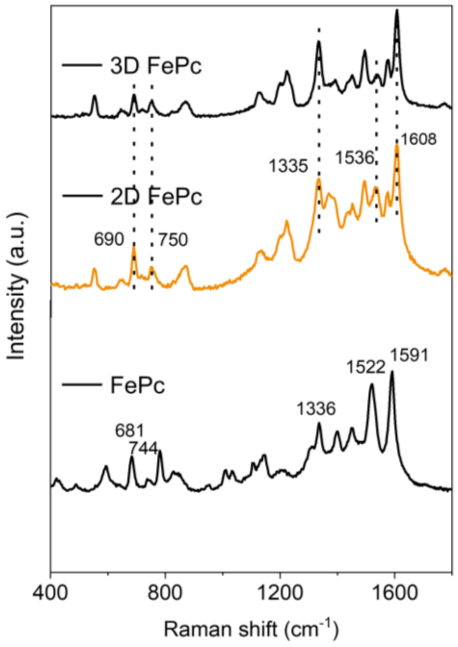

Figure S3. Raman spectra for for 3D FePc, 2D FePc and FePc.

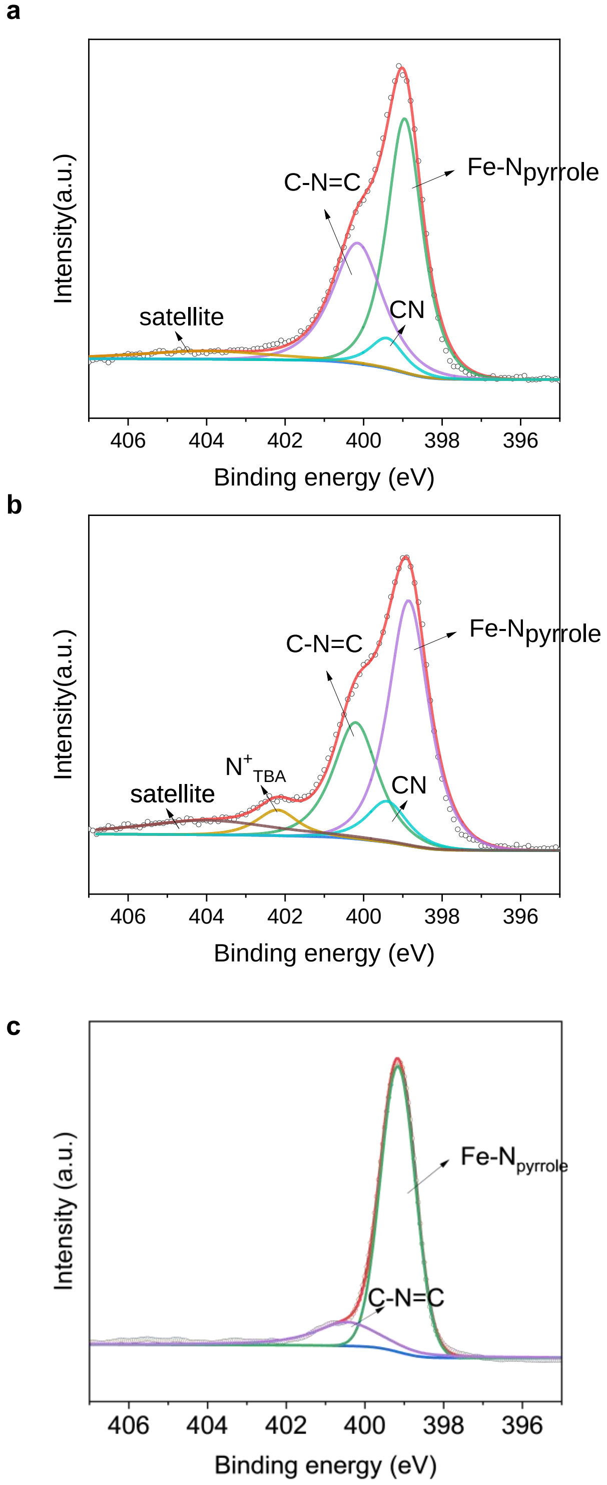

Figure S4. N 1s XPS of (a) 3D FePc, (b) 2D FePc and (c) FePc.

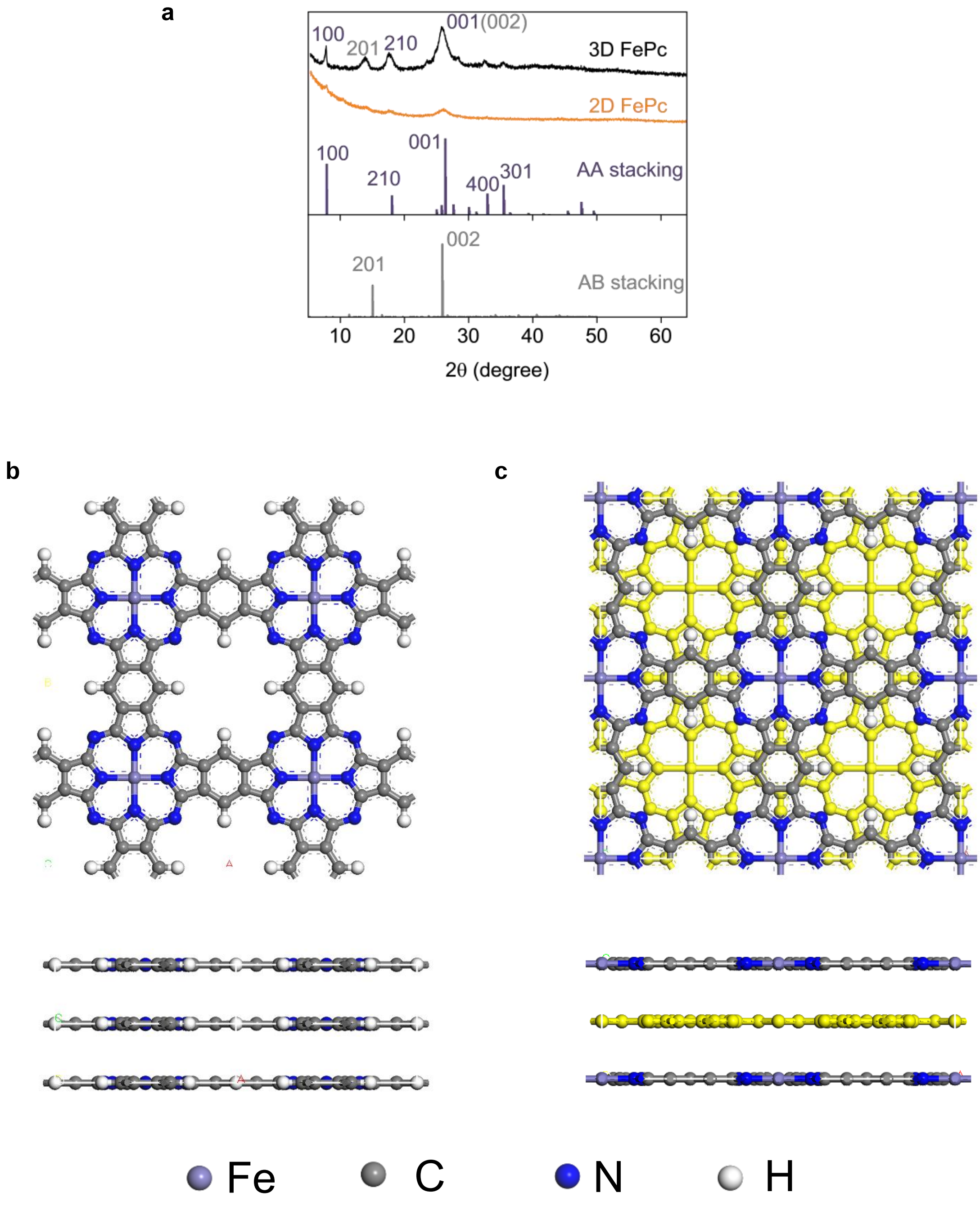

Figure S5. (a) Experimental and simulated PXRD patterns of 2D FePc and 3D FePc. (b) simulated AA stacking mode and (c) AB stacking.

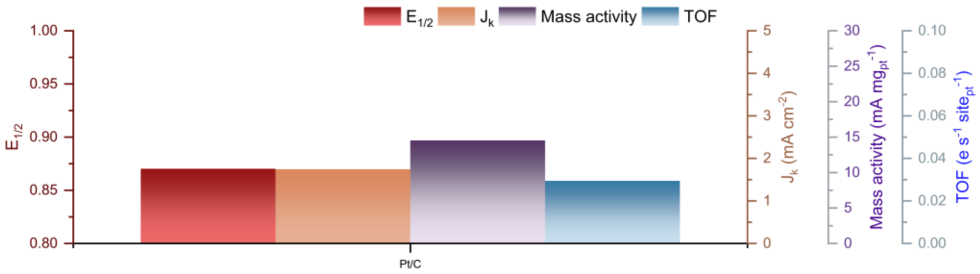

Figure S6. The ORR performance parameters for commercial Pt/C catalyst.

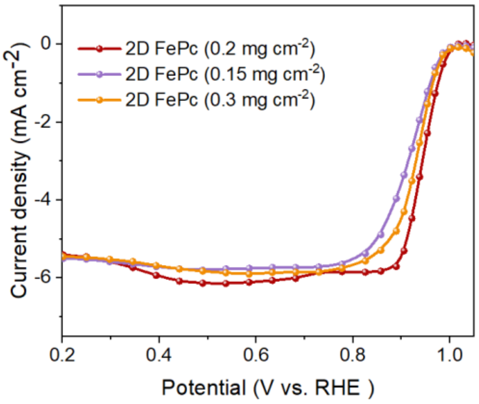

Figure S7. LSV curves for 2D FePc molecular catalyst with 0.3, 0.2 and 0.15 mg cm^{-2} loading.

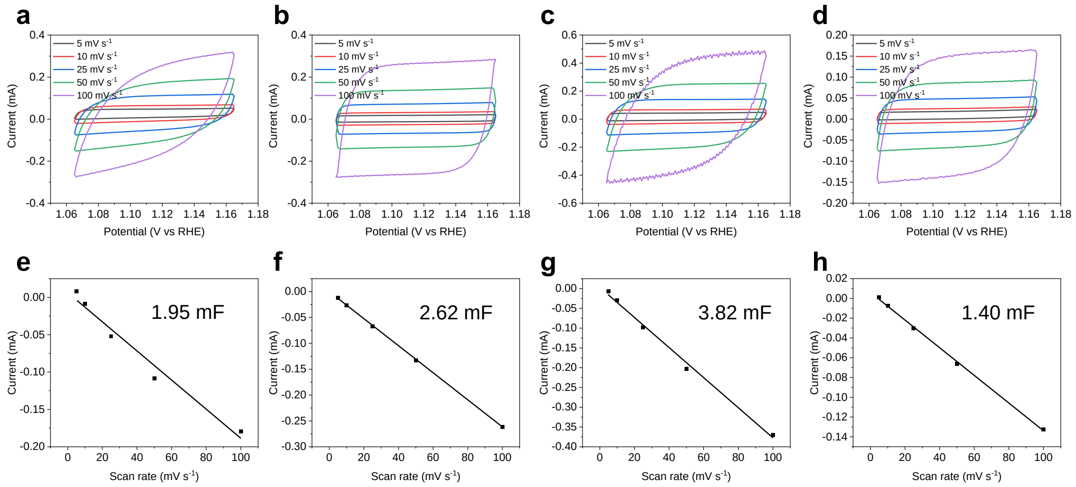

Figure S8. CV curves with different scan rate for (a) 3D FePc, (b) 2D FePc with 0.3 mg cm^{-2} loading, (c) 2D FePc with 0.2 mg cm^{-2} loading and (d) 2D FePc with 0.15 mg cm^{-2} loading. Capacitance (C_{dl}) of electric double layer for (e) 3D FePc, (f) 2D FePc with 0.3 mg cm^{-2} loading, (g) 2D FePc with 0.2 mg cm^{-2} loading and (h) 2D FePc with 0.15 mg cm^{-2} loading.

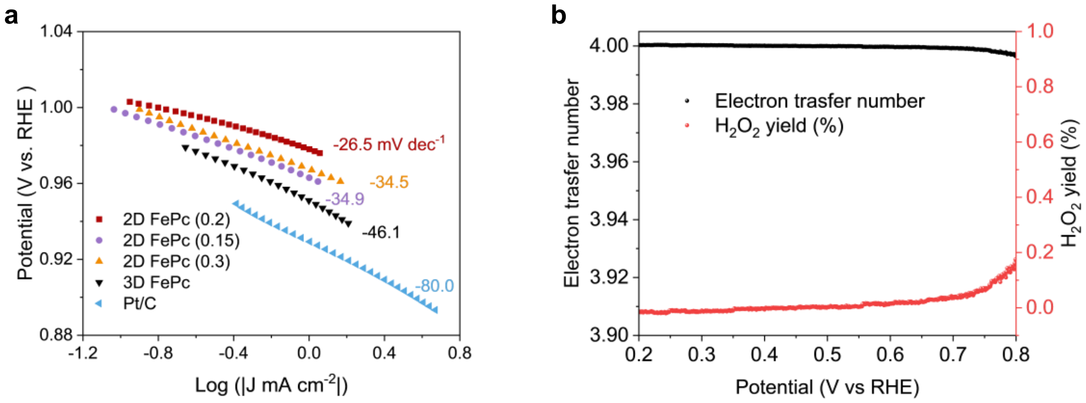

Figure S9. (a) The Tafel slope of 2D FePc catalyst and other counterparts. (b) ORR selectivity of 2D FePc catalyst (0.2 mg cm^{-2} loading), exhibiting exceptional 4e^{-} selectivity and an ultralow H_2O_2 yield.

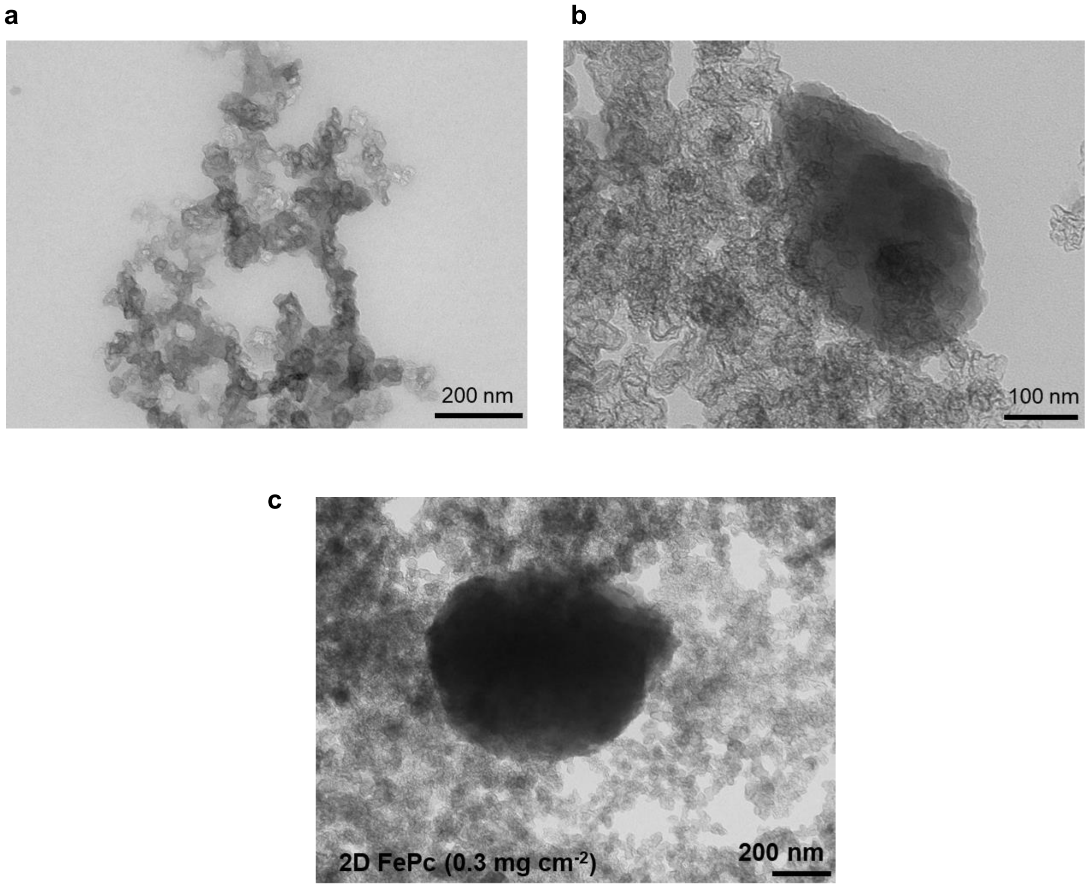

Figure S10. (a) TEM image of (a) carbon black and (b) 2D FePc molecular catalyst with 0.15 mg cm^{-2} loading. (c) 2D FePc molecular catalyst with 0.3 mg cm^{-2} loading.

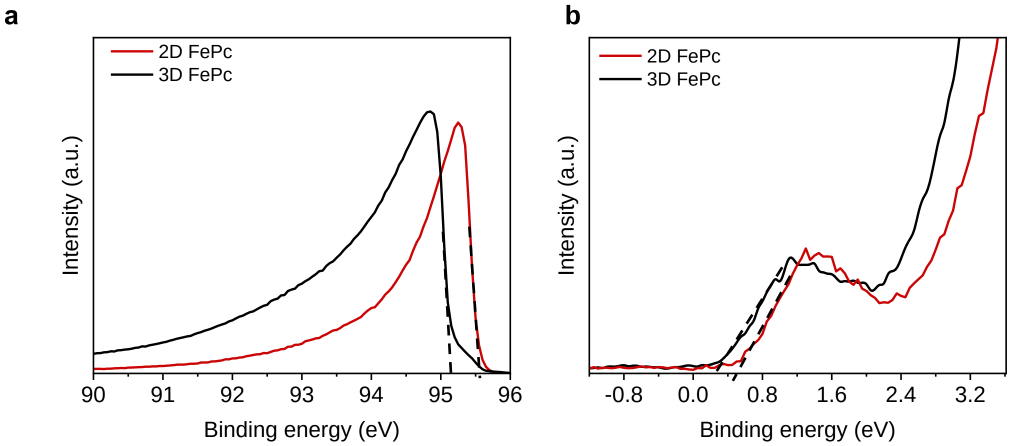

Figure S11. Synchrotron radiation photoelectron spectra (SRPES) for 3D FePc (black line) and 2D FePc (red line). (a) the secondary electron threshold. The work function is determined from a calculated with the formula (100 - Binding energy), which the value is 4.44 eV for 2D FePc and 4.84 eV for 3D FePc. (b) valence band. The value represents the energy gap between HOMO energy level and Fermi level, which the value is 0.27 eV for 2D FePc and 0.51eV for 3D FePc.

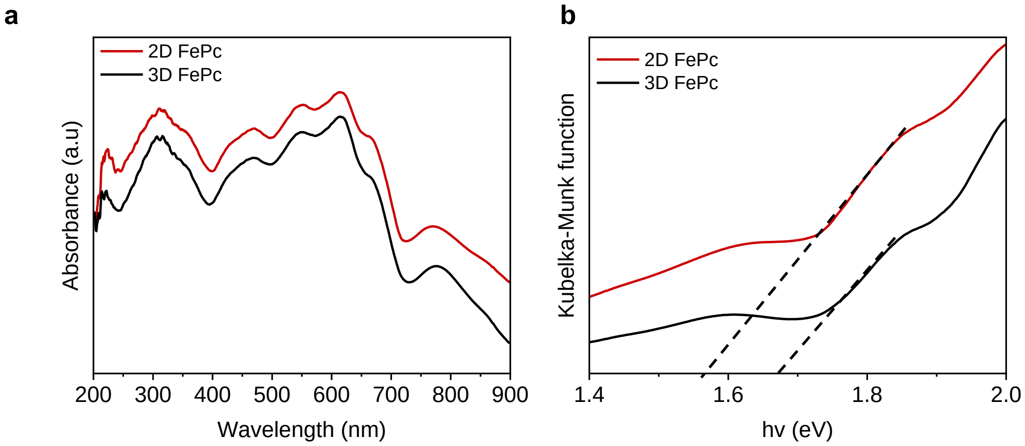

Figure S12. (a) Ultraviolet visible light spectrum (UV-VIS) for 3D FePc (black line) and 2D FePc (red line). The UV-Vis spectrum shows a typical peak distribution for FePc-based molecules composed of Q bands and B bands, as the results of π electron transition from HOMO to LUMO. (b) Kubelka-Munk function of UV-VIS as function of hv. Eg (optical HOMO-LUMO energy level gap) is determined from b, which the value is 1.58 eV for 2D FePc and 1.68 eV for 3D FePc.

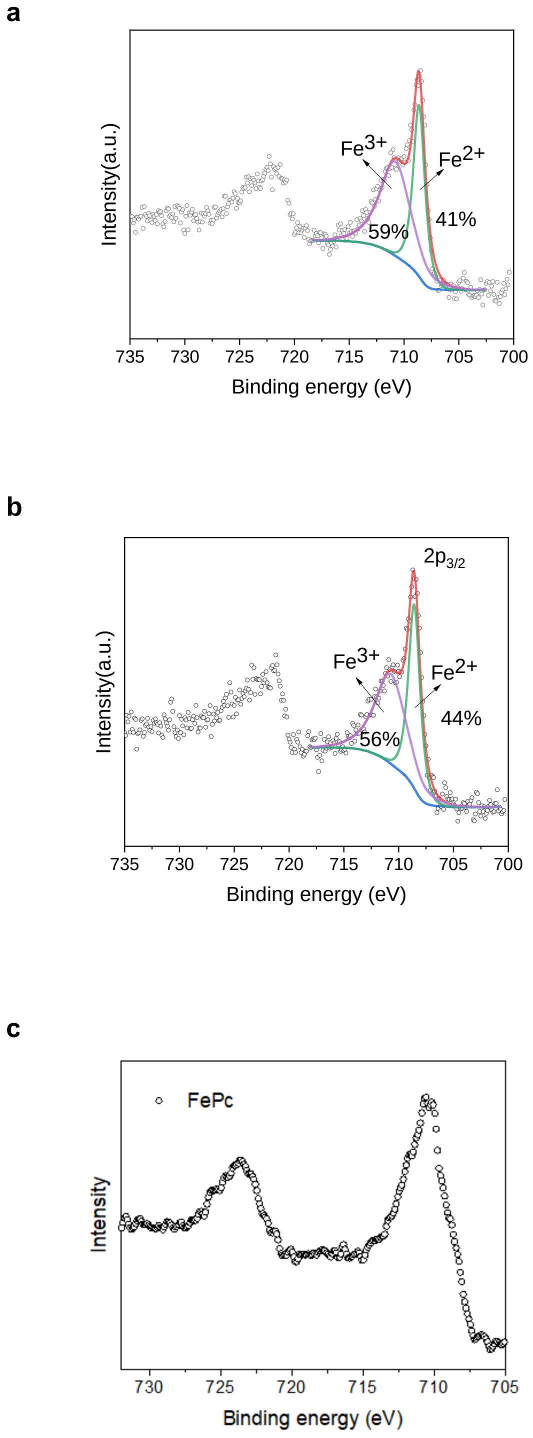

Figure S13. Fe 2p XPS for (a) 3D FePc, (b) 2D FePc and (c) FePc.

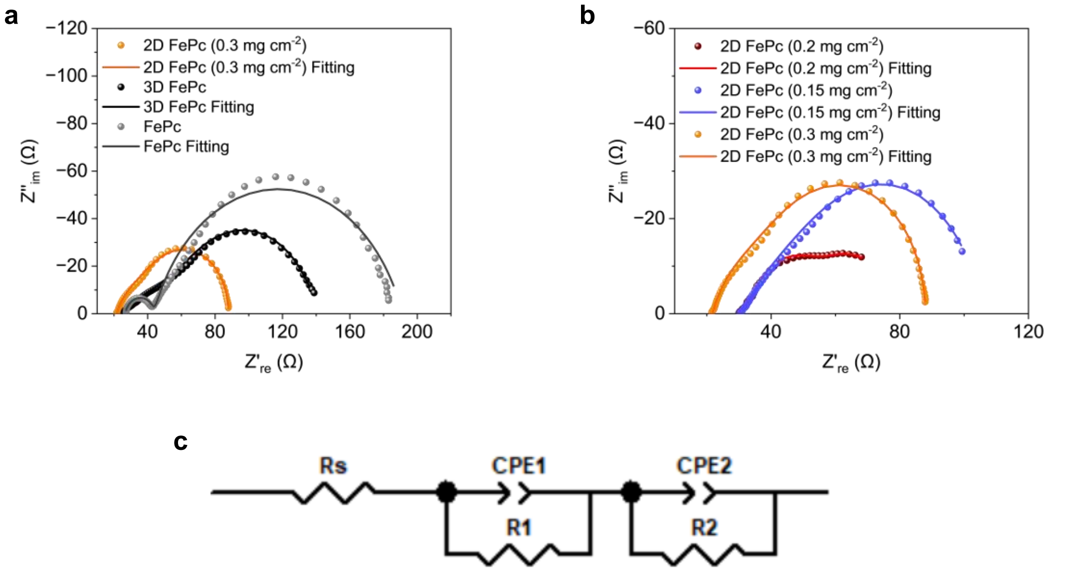

d

Catalysts	Rs	Rct (R1+R2)
**2D FePc ($0.2\ mg\ cm^{-2}$)**	33.46	43.94
**2D FePc ($0.15\ mg\ cm^{-2}$)**	30.55	77.54
**2D FePc ($0.3\ mg\ cm^{-2}$)**	21.8	66.2
**3D FePc**	25.06	119.96
**FePc**	26.59	164.53

Figure S14. EIS data and fitting plots for FePc series electrocatalysts at the potential of 0.935 V, illustrating the differences in charge transfer resistance and overall impedance behavior across the catalyst series.

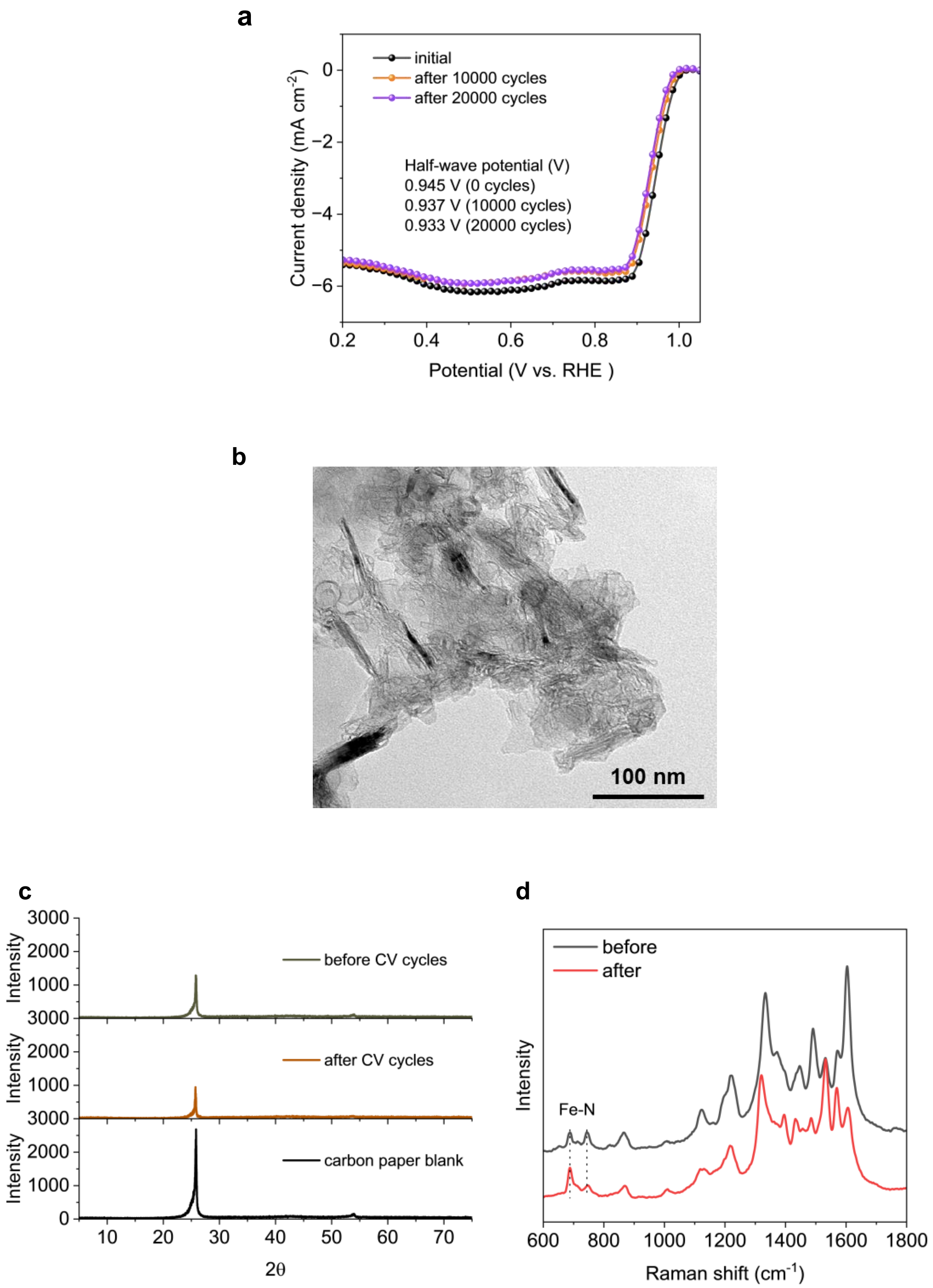

Figure S15. (a) 20000 cycles CV stability of 2D FePc; Morphological Stability after CV cycles (b); (c) XRD pattern before/after CV cycles (catalyst was dropped onto the carbon paper for tests); (d) operando Raman spectra.

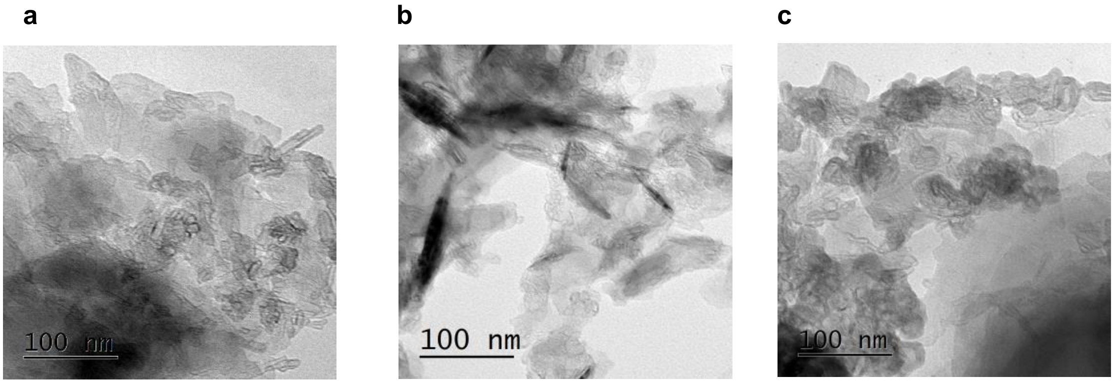

Figure S16. TEM image after (a) zinc-air battery and (b), (c) hydroxide exchange membrane fuel cells tests.

Figure S16a displays the TEM image after the Zn-air battery test. The morphology largely remains consistent with the pre-test condition, showing that 2D FePc maintains good dispersion and support, further confirming its structural robustness in a different electrochemical environment.

Figure S16b presents the TEM image of 2D FePc after the fuel cell test. The nanosheets remain well-dispersed and are still supported by carbon nanospheres, indicating that the hybrid structure is stable and effective in maintaining active sites within the EDL. Figure S16c shows a slightly altered region. In the lower right corner, we observe a less uniform mixture, where nanosheets appear less supported by the carbon substrate. However, the majority of the sample area still demonstrates a homogeneous distribution similar to that in Figure S16b. This local inhomogeneity could be attributed to the more demanding conditions of the fuel cell test or potential inconsistencies during the membrane electrode assembly (MEA) preparation.

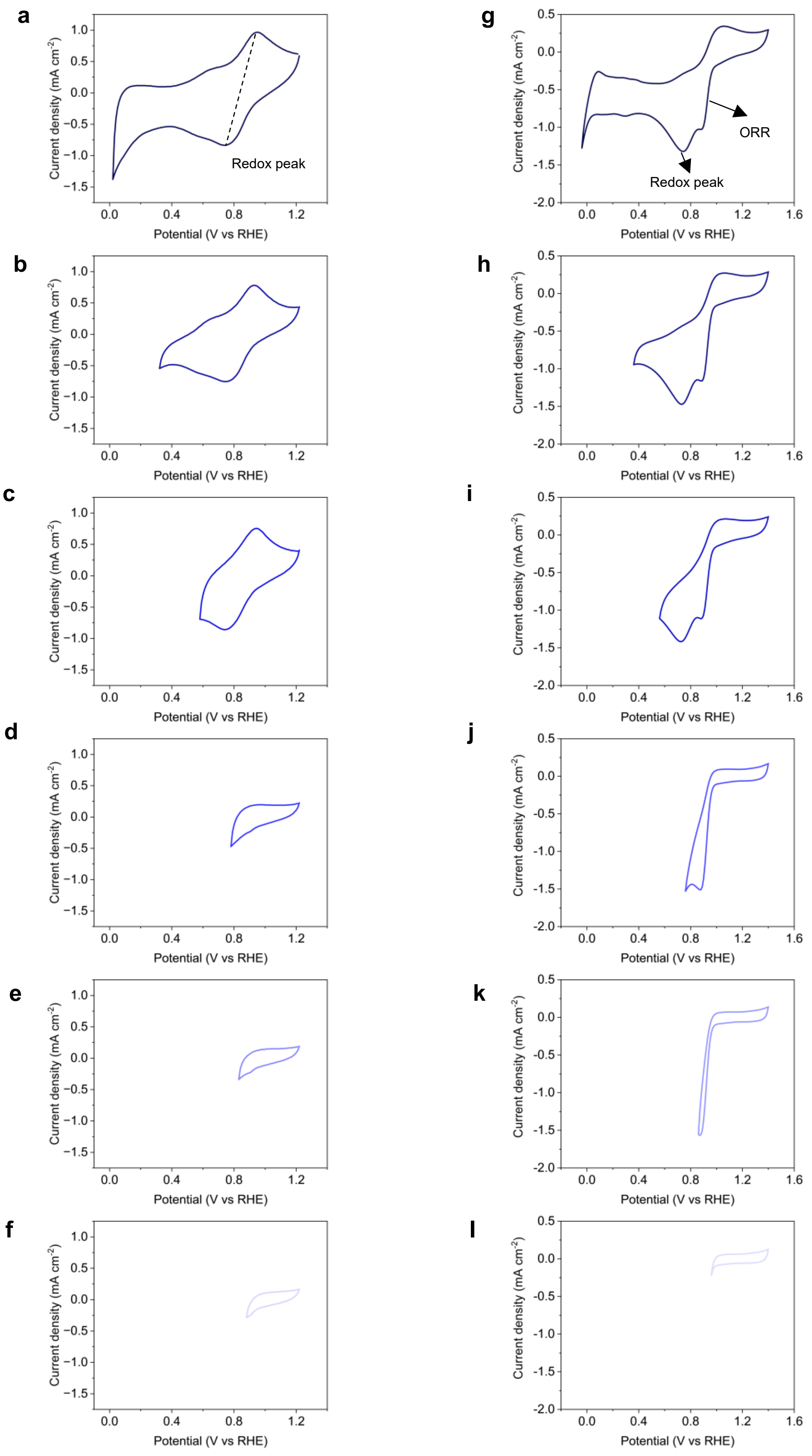

Figure S17. (a), (b), (c), (d), (e) and (f) CV of 2D FePc molecular catalyst under N_2-saturated 0.1 M KOH solution; (g), (h), (i), (j), (k) and (l) CV of 2D FePc molecular catalyst under O_2-saturated 0.1 M KOH solution.

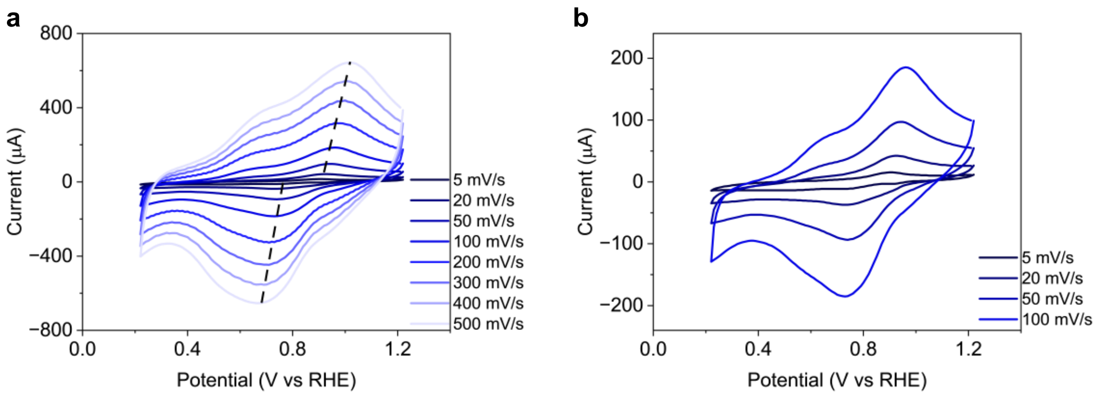

Figure S18. (a) CV of 2D FePc molecular catalyst with different scan rate under N_2-saturated and 0.1 M KOH solution; (b) Zoomed-in view of (a).

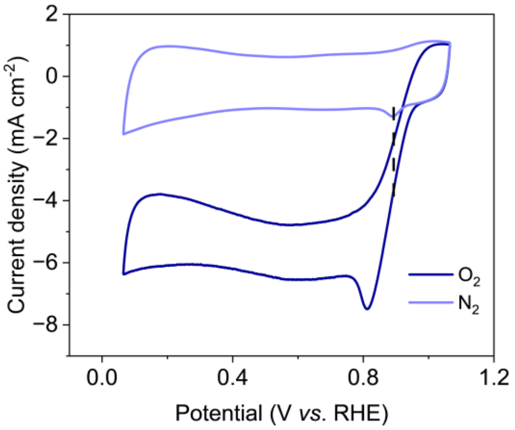

Figure S19. CV of 3D FePc catalyst under N_2-saturated or O_2-saturated 0.1 M KOH solution.

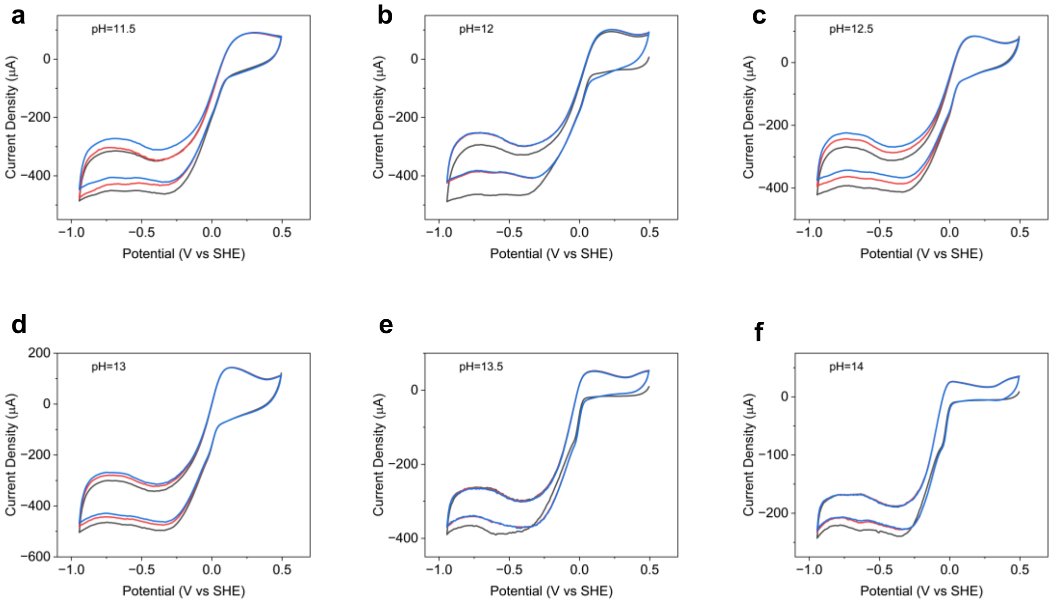

Figure S20. CV of 2D FePc molecular catalyst under O_2 condition at different pH with 1600 rpm rotation rate. (a) pH=11.5, (b) pH=12, (c) pH=12.5, (d) pH=13, (e) pH=13.5 and (f) pH=14.

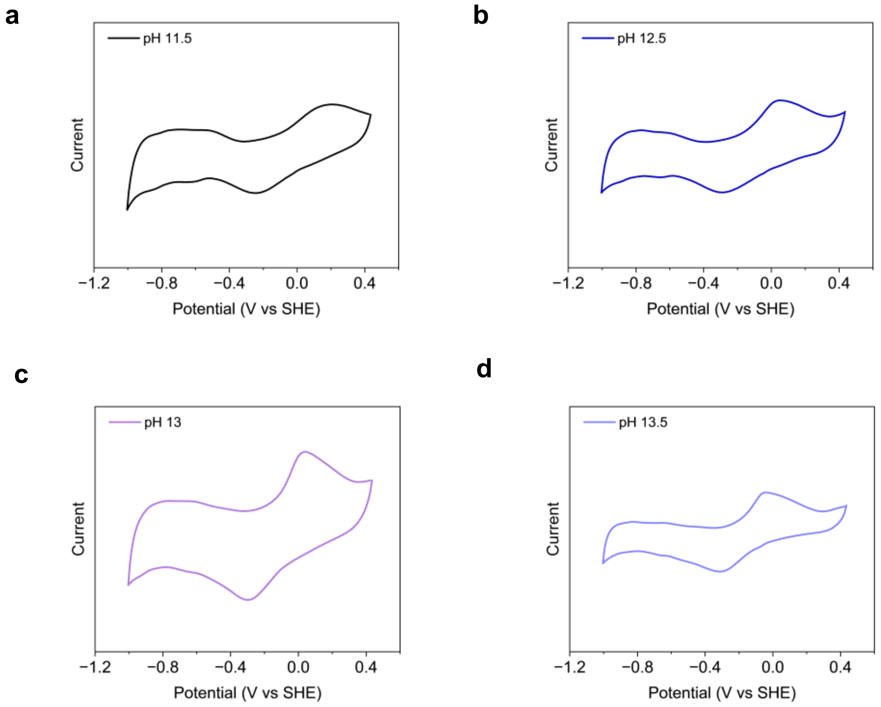

Figure S21. CV of 2D FePc molecular catalyst under N_2 condition at different pH with 1600 rpm rotation rate. (a) pH=11.5, (b) pH=12.5, (c) pH=13 and (d) pH=13.5.

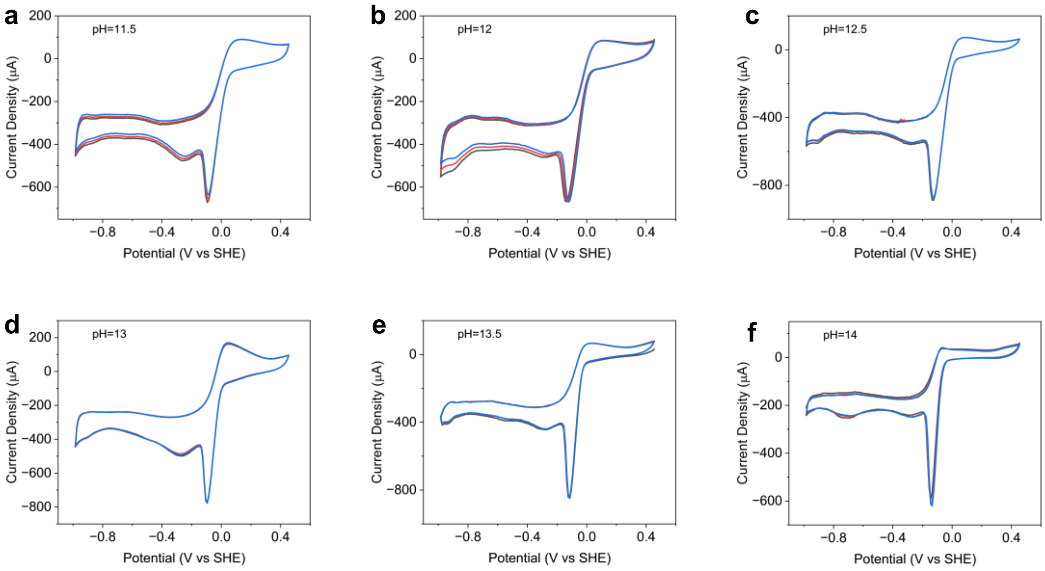

Figure S22. CV of 3D FePc molecular catalyst under O_2 condition at different pH with 1600 rpm rotation rate. (a) pH=11.5, (b) pH=12, (c) pH=12.5, (d) pH=13, (e) pH=13.5 and (f) pH=14.

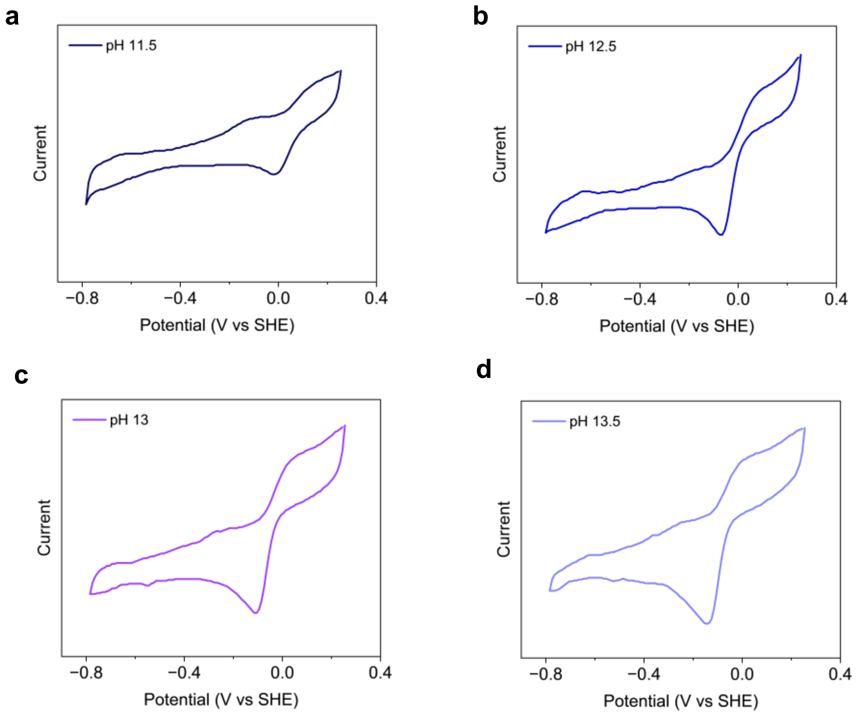

Figure S23. CV of 3D FePc molecular catalyst under N_2 condition at different pH with 1600 rpm rotation rate. (a) pH=11.5, (b) pH=12.5, (c) pH=13 and (d) pH=13.5.

Table S1. Summary of the ORR catalytic properties of reported Fe-N-C catalysts.

Catalyst	ORR performance (at 0.1 M KOH)		Zn-Air batteries performance		Ref
	$E_{1/2}$ (V vs. RHE)	stability	Peak power density	Battery stability	
2D FePc	0.945 V (loading 0.2 mg cm^{-2})	20000 cycles	165 mW cm^{-2} (loading 0.33 mg cm^{-2})	350 hours at 5 mA cm^{-2}	**This work**
FeSAs/NSC	0.87 V (loading 0.2 mg cm^{-2})	5000 cycles	NA	NA	J. Am. Chem. Soc. 2019, 141, 51, 20118–20126
pf SAC-Fe-0.2	0.91 V (loading 0.255 mg cm^{-2})	6000 cycles	126.83 mW cm^{-2} (loading 0.5 mg cm^{-2})	5 hours at 100 mA cm^{-2}	Sci. Adv. 5, eaaw2322
FeAB–O	0.90 V (loading 0.2 mg cm^{-2})	2.8 hours I-t discharge	NA	NA	Nat. Commun. 2020, 11, 4173
Fe/N-G-SAC	0.89 V (loading 0.6 mg cm^{-2})	10000 cycles	120 mW cm^{-2} (loading 1.0 mg cm^{-2})	NA	Adv. Mater. 2020,32,2004900
FeN x@Fe/Fe3C	0.899 (loading 0.7 mg cm^{-2})	5000 cycles	NA	NA	J. Am. Chem. Soc. 2016, 138, 10, 3570-3578.
2D Fe−N−C	0.91 V (loading 0.4 mg cm^{-2})	12 mV decay after 6000 cycles	160 mW cm^{-2} (loading 0.5 mg cm^{-2})	135 hours at 5 mA cm^{-2}	ACS Materials. Lett. 2020, 2, 1, 35–41
CAN-Pc(Fe/Co)	0.84 V (loading 0.02 mg cm^{-2})	3.3 hours I-t discharge	88 mW cm^{-2} (loading 0.1 mg cm^{-2})	60 hours at 5 mA cm^{-2}	Angew. Chem. 2019, 131, 14866 - 14872
$Fe_{0.5}Co_{0.5}$Pc-CP NS@G	0.927 V (loading 0.25 mg cm^{-2})	20000 seconds I-t discharge	180 mW cm^{-2}	60 hours at 10 mA cm^{-2}	J. Mater. Chem. A, 2019,7, 3112-3119
Fe-NCCs	0.82 V (loading 0.1 mg cm^{-2})	5000 cycles	70 mW cm^{-2} (loading 1.0 mg cm^{-2})	60 hours at 5 mA cm^{-2}	ACS Appl. Energy. Mater. 2018, 1, 9, 4982–4990
S,N-Fe/N/C-CNT	0.85 V (loading 0.6 mg cm^{-2})	10000 cycles	102.7 mW cm^{-2} (loading 1.25 mg cm^{-2})	NA	Angew. Chem. 2017, 129, 625 –629
Fe–N-SCCFs	0.883 V (loading 0.24 mg cm^{-2})	10000 cycles	300 mW cm^{-2} (loading 1 mg cm^{-2})	10 hours at 50 mA cm^{-2}	Nano lett. 2017, 17, 3: 2003-2009.

FeN_x-PNC	0.86 V (loading 0.14 mg cm^{-2})	20 mV decay after 10000 cycles	278 mW cm^{-2} (loading NA)	40 hours discharge-charge cycling at 5 mA cm^{-2}	ACS nano. 2018, 12(2): 1949-1958.
Fe-ISA/NC	0.896 V (loading 0.510 mg cm^{-2})	15000 cycles	NA	NA	Adv. Mater. 2018, 1800588
FeCo-MHs	0.95 V (loading 0.2 mg cm^{-2})	10 hours I-t discharge	319.7 mW cm^{-2} (loading 1.0 mg cm^{-2})	2000 hours at 10 mA cm^{-2}	J. Am. Chem. Soc. 2023, 145, 39, 21273–21283
Fe-ISAs/CN	0.900 V (loading 0.408 mg cm^{-2})	5000 cycles	NA	NA	Angew. Chem. 2017, 56(24): 6937-6941.
Fe-N-HPe	0.910 V (loading 0.255 mg cm^{-2})	8 mV decay after 10000 cycles	164.8 mW cm^{-2} (loading 1.0 mg cm^{-2})	140 hours at 10 mA cm^{-2}	J. Mater. Chem. A. 2021, 9, 9761-9770
Fe50-N-C-900	0.92 V (loading 0.1 mg cm^{-2})	5.5 hours I-t discharge	NA	NA	Small. 2018, 14, 1703118
FeN_4-PN	0.91 V (loading 0.2 mg cm^{-2})	NA	NA	NA	ACS Catal. 2021, 11, 6304−6315
Fe–NC–SAC	0.90 V (loading 0.6 mg cm^{-2})	5000 cycles	NA	NA	Nat Commun. 2019, 1, 1278
Fe-NC-S	0.88 V (loading 0.2 mg cm^{-2})	NA	NA	NA	Chem.2020.10.027
FePc/CoPc HS	0.879 V (loading 0.05 mg cm^{-2})	35% decay after 2.8 hours I-t discharge	128 mW cm^{-2} (loading 1 mg cm^{-2})	NA	Adv. Funct. Mater. 2020, 2005000

Table S2. Summary of the start-of-art ORR catalyst.

Catalyst	ORR performance		Zn-Air batteries / H_2-O_2 fuel cell performance		Ref
	$E_{1/2}$ (V vs. RHE)	stability	Peak power density in Zn-Air batteries	Peak power density in H_2-O_2 fuel cell	
2D FePc	0.945	20000 cycles	165 mW cm^{-2}	200 mW cm^{-2}	**This work**
Fe-SAs/MPC	0.927	30000 cycles	232 mW cm^{-2}	NA	*Angew. Chem. Int. Ed.* 2025, e202501307
FeCo-N_3O_3@C	0.936	10000 cycles	143 mW cm^{-2}	NA	*Nat. Synth* 3, 878–890 (2024)
Fe-N_2-Fe DAC	0.91	40000 cycles	169.8 mW cm^{-2}	NA	*Angew. Chem. Int. Ed.* 2024, 63, e202408914
HESA	0.87	1000 cycles	207 mW cm^{-2}	NA	*Nat Sustain* 6, 816–826 (2023)
Co_{SA}Ni-NCNT/CNF	0.86	50h i-t test	132.23 mW cm^{-2}	NA	*Adv. Energy Mater.* 2024, 14, 2400347
T-Fe SAC	0.91	10000 cycles	199 mW cm^{-2}	NA	*Angew. Chem. Int. Ed.* 2024, 63, e202319370
PtSb IMC	0.884	20000 cycles	NA	1.57 W cm^{-2}	*Natl Sci Rev*, 2024, Vol. 11, nwae233
Surf-IMPtFe NWs	0.956	30000 cycles	NA	1.4 W cm^{-2}	*Sci. Adv.* 10, eado4935 (2024)
PtFe@Fe_{SAs}-N-C	0.872	30000 cycles	NA	1.24 W cm^{-2}	*Nat. Commun.* 2024, 15, 5990
PtMg/C-31	0.87	30000 cycles	NA	1.575 W cm^{-2}	*Nat. Commun.* 2024, 15, 7034
$L1_0$–PtNi–Ni/ZrO_2 RMSI	0.94	400000 cycles	NA	1.52 W cm^{-2}	Angew. Chem. Int. Ed. 2024, 63, e202400751
$L1_0$-PtCoIn@Pt	0.938	1200000 cycles	NA	1.99 W cm^{-2}	Angew. Chem. Int. Ed. 2025, e202501805

Reference

1. Wang Y, Wang M, Chen T, Yu W, Liu H, Cheng H*, et al.* Pyrazine-linked Iron-coordinated Tetrapyrrole Conjugated Organic Polymer Catalyst with Spatially Proximate Donor-Acceptor Pairs for Oxygen Reduction in Fuel Cells. *Angewandte Chemie International Edition* 2023, **62**(47)**:** e202308070.
2. Peng J, Liu Y, Lv H, Li Y, Lin Y, Su Y*, et al.* Stoichiometric two-dimensional non-van der Waals AgCrS2 with superionic behaviour at room temperature. *Nature Chemistry* 2021, **13**(12)**:** 1235-1240.
3. Wang Y, Zhou T, Ruan S, Feng H, Bi W, Hu J*, et al.* Directional manipulation of electron transfer by energy level engineering for efficient cathodic oxygen reduction. *Nano Letters* 2022, **22**(16)**:** 6622-6630.
4. Fei H, Dong J, Arellano-Jiménez MJ, Ye G, Dong Kim N, Samuel EL*, et al.* Atomic cobalt on nitrogen-doped graphene for hydrogen generation. *Nature communications* 2015, **6**(1)**:** 1-8.
5. Mei Zy, Zhao G, Xia C, Cai S, Jing Q, Sheng X*, et al.* Regulated high-spin state and constrained charge behavior of active cobalt sites in covalent organic frameworks for promoting electrocatalytic oxygen reduction. *Angewandte Chemie* 2023, **135**(27)**:** e202303871.
6. Wang Y, Zhang D, Liang X, Shehzad M, Xiao X, Zhu Y*, et al.* Improving fuel cell performance of an anion exchange membrane by terminal pending *bis*-cations on a flexible side chain. Journal of Membrane Science 2020, 595, 117483.
